# Chemical Gas Sensors: Recent Developments, Challenges, and the Potential of Machine Learning—A Review

**DOI:** 10.3390/s21082877

**Published:** 2021-04-20

**Authors:** Usman Yaqoob, Mohammad I. Younis

**Affiliations:** Department of Physical Science and Engineering, King Abdullah University of Science and Technology, Thuwal 23955-6900, Saudi Arabia; usman.yaqoob@kaust.edu.sa

**Keywords:** chemiresistive and FET sensors, carbon materials, 2D TMDCs, metal/metal oxide, density function theory (DFT), selectivity, drift compensation, machine learning, data processing, feature extraction, smart sensors, smart breath analyzers, PCA, classifiers

## Abstract

Nowadays, there is increasing interest in fast, accurate, and highly sensitive smart gas sensors with excellent selectivity boosted by the high demand for environmental safety and healthcare applications. Significant research has been conducted to develop sensors based on novel highly sensitive and selective materials. Computational and experimental studies have been explored in order to identify the key factors in providing the maximum active location for gas molecule adsorption including bandgap tuning through nanostructures, metal/metal oxide catalytic reactions, and nano junction formations. However, there are still great challenges, specifically in terms of selectivity, which raises the need for combining interdisciplinary fields to build smarter and high-performance gas/chemical sensing devices. This review discusses current major gas sensing performance-enhancing methods, their advantages, and limitations, especially in terms of selectivity and long-term stability. The discussion then establishes a case for the use of smart machine learning techniques, which offer effective data processing approaches, for the development of highly selective smart gas sensors. We highlight the effectiveness of static, dynamic, and frequency domain feature extraction techniques. Additionally, cross-validation methods are also covered; in particular, the manipulation of the k-fold cross-validation is discussed to accurately train a model according to the available datasets. We summarize different chemresistive and FET gas sensors and highlight their shortcomings, and then propose the potential of machine learning as a possible and feasible option. The review concludes that machine learning can be very promising in terms of building the future generation of smart, sensitive, and selective sensors.

## 1. Introduction

Today, the rapid expansion in automobiles and industries emerge as a serious threat to the environment and human health safety. Therefore, along with the strict implementation of industrial waste regulations, the demand for highly reliable and accurate sensors has become essential for human survival [1,2,3,4,5]. Over the past decades, several gas-sensing mechanisms have been reported, including resistive/chemiresistive [6], electrochemical [7,8] (amperometric and potentiometric), work function (Schottky diode, metal–oxide–semiconductor field-effect transistors (MOSFET) [9,10], etc.), optical (surface plasmon resonance (SPR)), and surface acoustic [11,12,13,14]. Among them, resistive type gas sensors have been extensively studied due to their small size, low power consumption, cheap and simple fabrication process [10]. To date, various materials have been developed and used for resistive-type sensing, such as carbon materials (graphene/reduced graphene oxide (rGO) [15,16,17,18], carbon nanotubes (CNTs) [9,19], carbon and graphene dots [20,21,22], three-dimensional (D) graphene [23,24], etc.), 2D transition metal dichalcogenides (TMDCs) [25,26], metal oxides (zinc oxide (ZnO) [27], tin oxide (SnO_2_) [28], titanium oxide (TiO_2_) [29], tungsten oxide (WO_3_) [30], indium oxide (In_2_O_3_) [31,32], nickel oxide (NiO) [33], iron oxide (Fe_2_O_3_) [34,35,36], etc.) [6,37,38], and noble metal catalysts (pallidum (Pd), platinum (Pt), gold (Au), silver (Ag), aluminum (Al), rhodium (Rh), etc.) [15,39,40,41].

Carbon materials possess a higher surface area and have the capability for trace-level molecule detection at room temperature (RT). However, they are less selective and demonstrate a lower recovery rate due to their high binding energy with the gas molecules [42,43]. On the other hand, metal oxides (MOx) are good candidates to detect a wide range of gas molecules at higher concentration levels with a relatively faster recovery rate. However, they require higher operating temperature (OT) to generate favorable oxygen adsorbents (O_2_^−^, O^−^, and O^2−^) on sensing surfaces [44,45,46]. 

For environmental safety and better monitoring of human health, there is an urgent demand for the development of a sensor with trace-level molecule detection, minimum drift, high sensitivity, fast response/recovery, and excellent selectivity under different environments (dry and humid). With the aim to build a sensor having such properties, researchers have focused on synthesizing novel and sensitive sensing materials (SMs) using different techniques including surface morphology change/modification [22,27,30,46], doping [47], composition/hybridization [15,26,30,44,48,49], p–n junction formation [50,51], and core–shell structures [52,53,54,55]. SMs synthesized via these methods certainly have a key impact in improving sensor performances. For instance, recently Wu et al. [39] reported a high-performance NO_2_ sensor at RT using boron (B)- and nitrogen (N)-doped 3D reduced graphene oxide hydrogel (RGOH). In comparison with pure RGOH sensors, the B- and N-doped RGOH sensors exhibited 38.9 and 18 times higher responses toward 800 ppb NO_2_, respectively. Additionally, the fabricated sensors showed good linearity, reversibility, fast response/recovery, and impressive selectivity. The higher sensing performances from B- and N-doped RGOH sensors at RT was ascribed to several factors, including the doping effects of B and N, 3D porous rGO architecture with the enlarged surface-to-volume ratio, pore filling, charge hopping, abundant disorder, oxygenated groups, and high electron mobility [39]. On the contrary, metal oxide gas sensors, which require higher operating temperature, can also show good sensing performance via metal catalyst doping and morphological modification. Recently, Sanger et al. [56] presented a highly sensitive transparent NO_2_ sensor using aluminum (Al)-doped ZnO (AZO) hollow nanofiber synthesized via the sputtering method. Their sensors displayed maximum sensitivity at an OT of 250 °C with a detection range (DR) from 0.5 to 10 ppm. The high sensitivity of the transparent sensors was attributed to the higher surface area of the hollow nanofibers and the high impact frequency of the trapped NO_2_ gas inside the hollow compared to the solid counterpart nanofibers [56]. Similarly, Li et al. [57] reported Pd–Au nanoparticles (NPs) decorated on SnO_2_ nanosheets (NShs) for formaldehyde and acetone detection. They demonstrated the temperature-dependent selectivity of fabricated sensors toward formaldehyde and acetone. The results determined effective detection of acetone (@250 °C) and formaldehyde (@110 °C) with responses of 6.6 (acetone) and 4.1 (formaldehyde) towards 2 ppm concentration, and their corresponding detection limits were noted as 45 ppb and 30 ppb, respectively. The enhanced response was attributed to the chemical sensitization of Au, the electronic sensitization of Pd, and the synergistic effect of Pd–Au bimetallic NPs [57]. 

Additionally, bimetals/bimetal oxide core–shell structures were also studied to obtain better sensing outcomes [40,41]. Most recently, Xu et al. [58] studied formaldehyde (HCHO) detection using bimetal Ag@Pt core−shell nanostructures (NSs) decorated on ZnO nanowires (NWs) by an inkjet printing method. Optimized (with Pt_60_ and Ag_40_ atomic ratio) sensors demonstrated maximum response on an OT of 280 °C with DR varying from 120 ppb to 2 ppm. They described how Ag@Pt core−shell NPs play a vital role as a catalyst during the HCHO detection process by dramatically enhancing the oxidation of HCHO molecules on the ZnO (NWs) surface. As a result, more electron release brings a higher HCHO sensing response for the ZnO-based gas sensor [58].

Tuning the material properties through the mentioned techniques significantly improves almost all aspects of the sensor’s performance. However, realizing a sensor with excellent selectivity under humid conditions, trace level molecule detection, and good repeatability with no drift error due to aging and environment (temperature and humidity) is still a pressing challenge for the ongoing massive research in the field. 

It is well known that SMs tend to lose their properties with aging and environmental factors, and thus can show degradation in response and sensitivity towards unwanted gases. Therefore, along with the development of highly stable and sensitive materials, interdisciplinary studies are essential to overcome the limitations of SMs and to build promising and reliable sensor devices for their implementation in real-world applications. 

Machine learning is considered a favorable tool for developing smart devices with the ability to effectively tackle selectivity and drift problems. Selectivity is a major indicator in defining the sensor performance for both medical and environmental monitoring applications. For example, in the medical field, breath analyzers are used to detect a specific volatile organic compound (VOC) traces among thousands of VOCs in human breath for accurate disease diagnosis. Therefore, it is highly recommended that a breathalyzer should have the ability to detect the traces of specific VOC at certain concentrations with excellent selectivity for proper diagnosis. For environment monitoring, trace-level detection may not be required but excellent selectivity is still a key factor in characterizing the efficiency of a gas sensor. 

The past decades have witnessed considerable development in smart gas sensors and electronic noses (e-noses) using machine learning [59,60,61]. Machine learning mainly contributes to two major factors of a sensing device: drift compensation and selectivity. Over the past few decades, several methods have been investigated to address the drift error through univariate, multivariate, and machine learning [62,63,64,65]. Presently, machine learning is being used to tackle both drift compensation and selectivity [66,67,68]. The machine learning technique involves data processing of sensor output, dimensionality reduction, and then training a system/network for the predictions [66]. Data processing aims to extract robust feature information from the dynamic sensor response, which can represent the unique “fingerprint” patterns for a particular gas to ensure the effectiveness of the subsequent pattern recognition algorithm [66,69]. A number of signal/data processing methods have been reported, such as steady-state (difference, relative difference, fractional difference, normalization, logarithmic difference, etc.), transient (integral and differential), and frequency domain (fast Fourier transform (FFT), continuous/discrete wavelet transform (C/DWT)) models [70]. Among them, the systems/models built on transient and frequency domain data processing/feature extraction methods reveal maximum output accuracy [69,70].

Dimensionality reduction, as the name suggests, is used to reduce the redundancy in high-dimensional preprocessed data. Principal component analysis (PCA), an unsupervised technique, has been extensively studied to improve sensor selectivity (see Appendix A). This is performed by reducing the signal dimension from hundreds of features to only one primary component having the most useful information to generate the unique signatures against the specific gas [69,71]. Then, the classifiers/networks are trained and tested on these unique patterns to evaluate the prediction accuracy of unseen preprocessed data. Pattern recognition algorithms are mostly developed using two different approaches: (1) linear classifier using statistical theory and (2) nonlinear classifier using neural network [59,72]. Among the commonly used classifiers discussed in the literature [59] are the linear discriminant analysis (LDA), *k*-nearest neighbors (KNN), classification and regression trees (CART), Gaussian naïve Bayes (NB), support vector machines (SVM), random forest (RF), and artificial neural networks (ANN). Recently, Salhi et al. [73] reported a smart early fire detection system using machine learning [73]. They collected 21,146 sample measurements from the sensors under usual and extreme conditions engendering risks and trained their classifiers. Among different supervised machine learning methods, their study estimated the largest accuracy score for CART (99.93%) and KNN (99.71%) for the given dataset. They further improved their accuracy rate to 100% by mean imputation, in which they computed the mean values in the training dataset and replaced them with missing data [73]. 

This review is divided into two parts: In the first part, we cover the recent developments and limitations of chemresistive gas sensors on the basis of three different kinds of sensing materials, namely, carbon allotropes, 2D transition metal dichalcogenides (TMDCs), and metal oxides (MOx). Different computational and experimental studies are explored to identify the key factors in providing maximum active locations for gas molecule adsorption such as bandgap tuning through different nanostructures, heteroatom doping, nano junction formations, and surface catalytic reaction through hybridization/composition, etc. Afterward, we highlight current limitations in sensor performance such as long-term stability and selectivity. Then, in the second part, machine learning is proposed as a potential approach to efficiently tackle these issues through pattern recognition algorithms. Further, we analyze the significance of different features (static, dynamic, and frequency domain) extracted from response curves of chemiresistive and field-effect transistor (FET) devices for the unique single point signature marker (dimensionality reduction) generation through principal component analysis (PCA) followed by accurate model training. Moreover, the significance of cross-validation techniques for accurate model training is also discussed, specifically manipulation of different k-fold CV methods to enhance the model training, even with the small number of datasets. Finally, the review also discusses recent progress in building highly selective chemiresistive and FET gas sensors and breath analyzers using machine learning. 

## 2. Chemical Gas Sensors: Achievements and Challenges

In this section, we focus on the SMs and discuss their structural properties for better sensing. Later, we highlight some computational studies on different kinds of SMs and the critical performance-enhancing factors. Finally, we discuss recent experimental advancements in three different kinds of gas sensors including carbon materials, 2D TMDCs, and MOx. 

**Figure 1:**
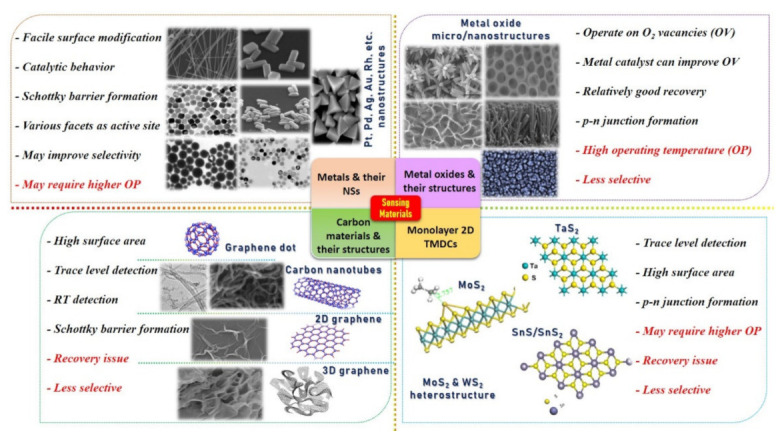
Different kinds of sensing materials with micro/nanostructures, their advantages (in black color), and limitations (in red color), indicating the need of hybridization/composition, doping, and p–n junction formation. In particular, the figure suggests that the decoration/doping of metal/hetroatom over the base materials will enhance the catalytic reaction for specific gases and form the charge accumulation depletion region to improve the sensing performances. Reproduced from multiple sources with permission from References [18,41,42,46,80,81,82,83,84,85,86,87,88,89,90,91]. Copyright 2016 Elsevier [18], copyright 2019 Elsevier [41], copyright 2016 Elsevier [42], copyright 2020 ACS [46], copyright 2020 [80], copyright 2019 Elsevier [92], copyright 2006 ACS [82], copyright 2007 ACS [83], copyright 2013 Elsevier [84], copyright 2014 Elsevier [85], copyright 2016 Elsevier [86], copyright 2018 ACS [87], copyright 2015 Elsevier [88], copyright 2014 Elsevier [89], copyright 2013 Nature [90], and copyright 2012 ACS [91].

The development of a chemical gas sensor with fast response/recovery time, maximum sensitivity, minimum aging drift, excellent selectivity, and repeatability are major research concerns and targets. Typically, a sensing material is considered a promising candidate if it possesses a high specific surface area and a highly reactive crystal facet/site for specific gas molecule adsorption with maximum charge transfer [6,20,25,44]. It is well documented that bulk metal/MOx materials change their physical and chemical properties entirely when synthesized in micro/nanostructures (NSs). Even by varying the nanostructured morphologies, dramatic change in material properties can be expected. For example, Miller et al. [74] investigated the various defects points on the SnO_2_ NPs and NW surfaces using the ultra-high spatial resolution scanning transmission electron microscopy (STEM) combined with cathodoluminescence (CL) system to interpret their role in manipulating the band gap of a nanostructure. They studied four different material samples and found that SnO_2_ NW decorated with NPs through sputtering reveals a higher number of defects and thus may enhance the sensor response. However, they proposed that more in-depth investigations under different temperatures and gases (oxidizing/reducing) are required to understand the effect of defects on gas sensing [45,74]. Tuning of the surface energy and altering the band gap both can be vital for providing active sites to gas molecules [32,75,76,77,78]. Therefore, considerable research works are conducted to develop and explore various micro- (from thick film to highly porous 3D hierarchical structure) and nanostructures (nanoparticles (NPs), nanorods (NRs), nanotube (NTs), nanowires (NWs), and nanocapsules (NCps), etc.) with the aim to build SMs with maximum specific surface area and higher number of active sites. Until now, numerous SMs have been synthesized using physical and chemical routes. Figure 1 displays different types of SMs and their micro/nanostructures with advantages and drawbacks. It also illustrates the significance of hybridization/composition formation to improve the overall performance. In particular, it suggests that the decoration of metal/metal oxide NSs on all other types of materials is extremely vital for higher catalytic reaction, depletion region formation (p–n junction and Schottky barriers), to improve response and selectivity. The detailed discussion on different heterostructures (p–n junction, Schottky barriers, and catalyst decoration) and their corresponding performance enhancement mechanisms is not within the scope of this review article. Interested readers are referred to recently published review articles [51,52,79].

Typically, SMs of a resistive-type gas sensor are composed of composite/hybrid materials, categorized as base and catalyst, and each has its role to play for better sensing performances. Base materials are usually responsible for providing a high surface area for gas molecule adsorption and a conductive pathway between two electrodes. This category includes microstructure thick films, thin films, porous films, 3D hierarchical structures, graphene, reduced graphene oxide, 3D graphene, and 2D transition metal dichalcogenides (TMDCs). On the other hand, metal/metal oxide catalysts, synthesized in nanostructures (NSs) and decorated over the base material, are used to enhance the reaction rate on the sensing surface for a specific gas. Therefore, a catalyst is an influential element in improving sensing performances, especially selectivity and response.

### 2.1. Computational and Experimental Research

#### 2.1.1. Identifying Key Performance-Enhancing Factors through Computational Analysis of the Graphene, TMDCs, and Metal Oxides Sensors

**Figure 2:**
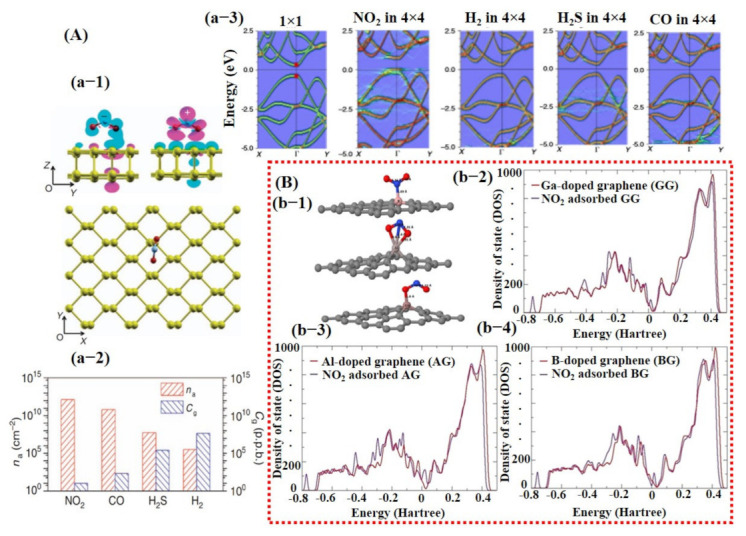
The computational investigation using DFT calculations: (**A**) DFT calculation for phosphorene: (a-1) shows adsorption of NO_2_ molecule on phosphorene surface with oxygen atoms pointing downward and the corresponding generated electron and hole clouds; (a-2) left axis indicates the histogram graph (red bars) with adsorption energies of different gases at fixed 20 ppb concentration; (a-3) displays the change in phosphorene band structure after adsorption of different gases including NO_2_, H_2_, H_2_S, and CO. A clear change on the energy level of conduction band can be observed with the NO_2_ adsorption on phosphorene surface, reproduced with permission from [94], copyright 2015 Nature. (**B**) DFT calculations of boron (B)-, aluminum (Al)-, and gallium-(Ga) doped graphene for NO_2_ detection: (b-1) illustrates a schematic for the NO_2_ molecule adsorption on all three kinds of graphene surfaces—clear and strong adsorption of NO_2_ molecule on Al-doped graphene can be seen (middle image); (b-(2–4)) show DOS for all kinds of graphene when exposed to NO_2_—Al-doped graphene displayed maximum change at Fermi energy level suggesting its higher sensing ability toward NO_2_ molecule (b-3). Reproduced with permission from [95], copyright 2016 Elsevier.

Over the decades, great efforts have been devoted to understanding the behavior of the gas molecules on different atomic/molecular sites of the SMs through density function theory (DFT) calculations. It is suggested that adsorption energy, charge transform, and distance between gas molecule and SM surface are highly significant in defining the performance ability of a sensor [25,26,93]. Gas molecule adsorption site/facet and landing orientation have a great influence on the adsorption energy and maximum charge transfer. High adsorption energy and charge transfer indicate high sensitivity and selectivity towards a specific gas. For instance, Cui et al. [94] studied the layer-dependent sensing performance of phosphorene using computational and experimental investigations. Maximum adsorption energy was noted for NO_2_ and was confirmed through variations in band structure, signifying higher sensitivity of phosphorene towards NO_2_ molecules (see Figure 2A). Nevertheless, even for NO_2_, the charge transfer was not very significant. Therefore, the decoration or doping of metal catalyst over the base SMs is needed to considerably raise the charge transfer and adsorption energy for specific gas [94]. Varghese et al. [95] investigated the gas-sensing properties of boron (B)-, aluminum (Al)-, and gallium (Ga)-doped graphene using DFT calculations. They discovered that B-doped graphene behaved more stable in humidity environment than that of Al- and Ga-doped graphene and displayed adsorption energies of −0.375 eV and −1.450 eV for NO and NO_2_ molecules, respectively. Furthermore, they calculated maximum adsorption energies for Al (−3.474 eV)- and Ga (−3.050 eV)-doped graphene towards NO_2_ molecules in a dry environment [95]. Figure 2B shows a schematic illustration of NO_2_ molecule adsorption on doped graphene along with its density of state (DOS) for all types of graphene. A clear change in charge distributions at Fermi energy level can be seen for all types of graphene after NO_2_ molecule adsorption. Likewise, Wang et al. [96] investigated the adsorption of CO molecules on pure graphene, N-doped graphene, and Al-doped graphene, and their results showed maximum charge transfer of 0.2346 eV between CO molecule and Al-doped graphene surface [96]. 

Similarly, iron (Fe)-doped single-layer and bi-layer graphene for CO, NO, SO_2_, and HCN adsorptions were explored by Tang et al. [97]. Fe-doped bi-layer graphene showed maximum adsorption for NO molecule and weakest for CO. Additionally, the semiconducting and magnetic behavior of SMs after gas molecule adsorption was also studied [97]. 

**Figure 3:**
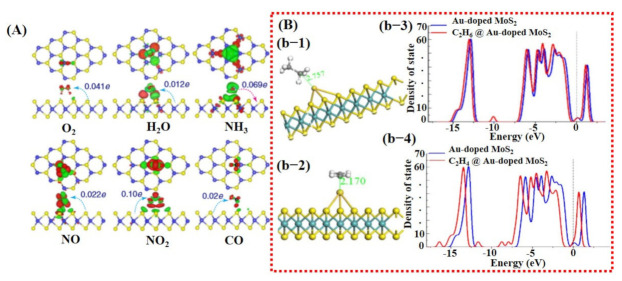
Computational analysis of MoS_2_ and Au-doped MoS_2_ for gas sensing using DFT calculations: (**A**) The charge transfer between pure MoS_2_ sheet and different target molecules; maximum charge transfer of 0.10 e can be seen for NO_2_, which is quite low, indicating the significance of heteroatom doping (reproduced with permission from [99], copyright 2013 Springer Nature). (**B**) The DFT results for Au-doped MoS_2_ sheet: (b-1) a schematic illustration of C_2_H_4_ and C_2_H_6_ molecule on Au-doped MoS_2_ with the corresponding bond length distance; (b-3,4) the DOS graphs for both the molecules before and after adsorption. A relatively larger change at Fermi energy level was found for C_2_H_4_, indicating better sensitivity of Au-doped MoS_2_ towards C_2_H_4_. Reproduced with permission from [80], copyright 2020 Front. Mater.

Besides graphene, 2D TMDCs and metal oxides have also been investigated using DFT calculations [26,98]. For example, a study by Yue et al. [99] considered the adsorption of different gas molecules including H_2_, O_2_, H_2_O, NH_3_, NO, NO_2_, and CO on pure MoS_2_ surface. They reported that all the gas molecules were weakly absorbed on MoS_2_ with less charge transfer, indicating the importance of doping/decoration of metal catalyst (see Figure 3A). They did not further continue their study with doping/decoration of a heteroatom. However, they proposed that the application of a perpendicular electric field promotes gas adsorption on the MoS_2_ surface [99]. More recently, Qian et al. [80] explored the effect of Au doping on 2D MoS_2_ monolayer sheets for C_2_H_6_ and C_2_H_4_ molecule detection. After Au doping, enhancement in adsorption energies and charge transfer between detection molecules and MoS_2_ monolayer was noted. The maximum adsorption energy and charge transfer were observed for the C_2_H_4_ molecule, finding it to be −0.952 eV and 0.309 e, respectively. Schematic diagrams are shown in Figure 3B of the Au@MoS_2_ surface with adsorbed gas molecules and DOS graphs. These techniques were not only studied in the computational domain but were also extensively investigated and verified through experiments. 

**Figure 4:**
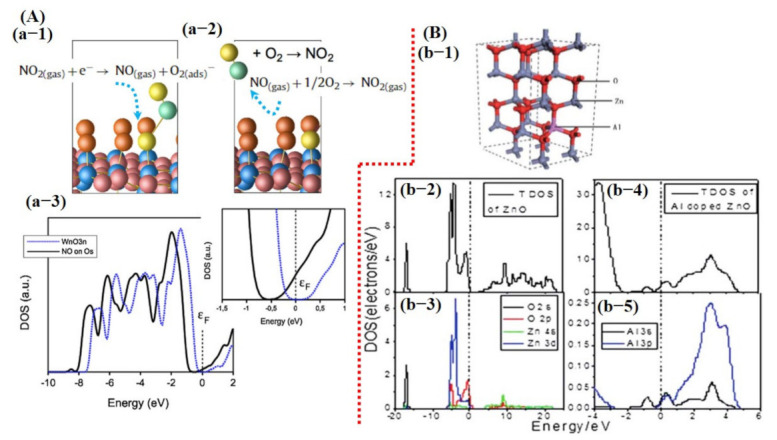
Computational study of metal oxide gas sensors: (**A**) NO_2_-sensing mechanism on WO_3_ surface and DOS graph before and after exposure of NO_2_: (a-1) The dissociation of NO_2_ molecule to NO when interacting with WO_3_ surface while leaving behind oxygen adsorbent. (a-2) The formation of NO_2_ from generated NO after reacting with one-half oxygen in the environment. This chain cycle significantly improves the oxygen adsorbent population consequently enhances the sensor response. (a-3) The DOS graph. A clear shift can be observed after NO exposure in the magnified image (reproduced with permission from [100], copyright 2014 Elsevier). (**B**) (b-1) A schematic image of Al-doped ZnO structure; (b-2) the change in Fermi energy level before and after Al doping in ZnO structure (reproduced with permission from [101], copyright 2013 RSC).

Saadi et al. [100] investigated the NO_2_ sensing mechanism on the WO_3_ surface, and they observed that the 001 facet of WO_3_ was more stable and suitable for NO_2_ sensing. Furthermore, they discussed the significance of oxygen vacancies on metal oxide surfaces and their role in the dissociation of NO_2_ molecules for the generation of more vacancies, and thus more active sites for the target molecule. DOS results displayed a change in charge distribution at Fermi energy level after NO_2_ adsorption, indicating the sensitivity of WO_3_ (001) surface towards NO_2_ molecules (Figure 2). The schematic illustration and reaction details/equations for NO_2_ dissociation in NO and again forming to NO_2_ are shown in Figure 4A. Bai et al. [101] reported both computational and experimental results of Al-doped ZnO NSs for CO sensing. Their experimental and simulated works were well matched and showed enhanced sensing performance towards CO when ZnO NSs were doped with Al (Figure 4B).

#### 2.1.2. Experimental Progress in Carbon Materials, 2D TMDCs, and MOx-Based Chemical Sensors

**Figure 5:**
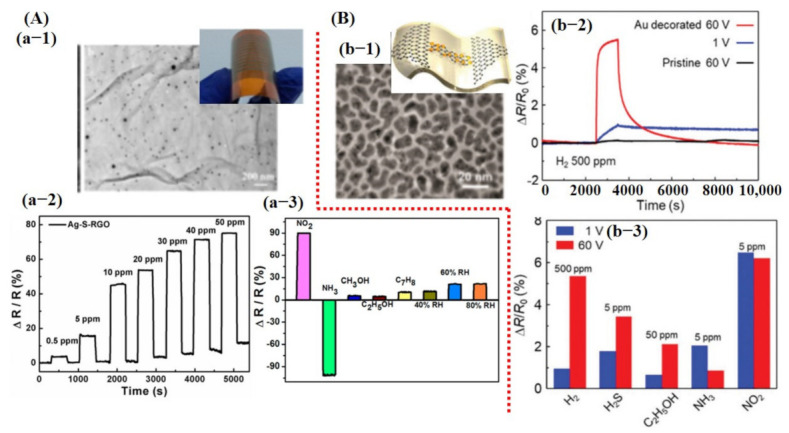
Experimental results of graphene-based gas sensors: (**A**) Ag NPs decorated on sulfonated reduced graphene oxide (Ag-S-rGO) and their sensing performance for NO_2_ and NH_3_ detection: (a-1) SEM image of Ag-S-rGO. Uniformly distributed Ag NPs can be seen on S-rGO surface. (a-2) Sensor response graph towards different concentrations of NO_2_ gas indicating good sensitivity, fast response/recovery, and linear change in sensor response at different concentrations. (a-3) Response graph to different gases/compounds including 100 ppm NO_2_, 1 mL (13 ppt) of NH_3_, 1 mL (46 ppt) of methanol, 1 mL (32 ppt) of ethanol, 1 mL (18 ppt) of toluene, 40% RH, 60% RH, and 80% RH. Reproduced with permission from [103], copyright 2014 ACS. (**B**) The results for Au @ graphene to detect H_2_ gas: (b-1) SEM image of Au@graphene; inset shows the schematic image of the device. (b-2) The response of pristine graphene and Au@graphene to 500 ppm H_2_ gas when 1 V and 60 V DC was applied. Sensor shows the maximum response when 60 V was applied, indicating the importance of biasing voltage. (b-3) A cross-sensitivity graph, suggesting very poor selectivity of the fabricated sensor. Reproduced with permission from [104], copyright 2019 RSC.

Experimentally, numerous research works have been presented on various materials and methods to improve gas sensing performance. Carbon materials including 0D carbon dot, 1D carbon nanotubes (CNTs), 2D graphene, reduced graphene oxide (rGO), and 3D graphene foam/crumpled graphene have been extensively studied for gas sensing due to their high surface area, high electron mobility at room temperature (RT), and high stability and mechanical flexibility [9,17,20,22,23,24,102]. Pure graphene and rGO have proven to be good candidates for trace level molecule detection at RT but exhibit higher response time with no recovery to baseline [42,43,102]. As computational analysis suggests, doping of heteroatom and decoration of metal catalyst dramatically improve the adsorption energy and charge transfer. Therefore, experimentally, those techniques were extensively investigated and proven to be highly promising. For example, sulfur-doped rGO sheets decorated with Ag NPs (10–20 nm) showed an excellent response at RT with full recovery. Fabricated Ag-S-rGO sensor revealed a good response towards NO_2_ and NH_3_ with a small response value to some other gasses. Additionally, the sensor displayed 45% sensitivity towards 50 ppm NO_2_ concentration with a response/recovery time of 12 s/20 s [103] (see Figure 5A). 

Biasing voltage affects the carrier concentration on the sensing surface and it is understood that modulating biasing voltage may play a vital role in better sensing performance. A similar study was carried out by Kim et al. [104]. They developed Au nanocubes (NCs) decorated on a graphene channel for enhanced H_2_ detection. It was found that after applying 60 V DC, the deposited Au layer morphology changed to the NCs and enabled better gas sensing at RT. Fabricated sensors displayed noteworthy improvement towards H_2_ detection after decoration with Au catalyst. However, their sensor did not reveal good selectivity and showed a significant response, even at lower concentrations for other gases [104] (see Figure 5B).

To further improve the selectivity, installation of a separate filter membrane is considered a potential approach for selective detection of a target analyte. In this technique, unwanted gases/chemical mixtures are pre-separated via a filter membrane with a specific pore size to allow the target analyte to pass through and reach the sensor surface. Materials with microporous surfaces such as zeolite, graphene, polymers, and metal−organic frameworks can be widely tuned according to the target analyte size, and are thus considered promising candidates for filter membrane development [105]. Most recently, Jarig et al. chemically synthesized a pore-sized tuned GO filter membrane for selective detection of various VOCs/VSCs. Their fabricated holey GO sheet with various pore sizes displayed superior cross-selectivity to CH_3_COCH_3_ (0.46 nm), C_2_H_5_OH (0.45 nm), C_7_H_8_ (0.59 nm), and H_2_S (0.36 nm). The synthesized holey GO filter membrane was placed over sensing material (PdO–WO_3_ nanosheets) that act as a molecular sieving layer to selectivity pass target analyte according to the pore size distribution on its surface. They claimed that a pore size-tuned GO porous layer is promising for designing low-cost and highly efficient gas sensors with outstanding selectivity [105]. However, these filter membranes might be restricted to very small size analyte detection as the bigger pores may allow for diffusion of several other gases/chemicals with similar diameters, resulting in poor selectivity.

**Figure 6:**
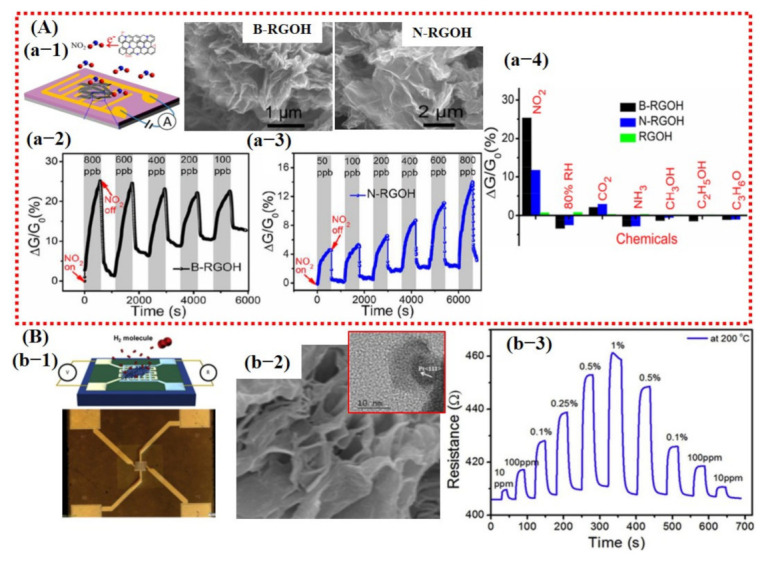
Representation of the sensing performances of 3D graphene: (**A**) Sensing results of B- and N-doped RGOH: (a-1) Schematic diagram of the fabricated sensor device with SEM images of highly porous B- and N-doped graphene. (a-2,3) The response of B-RGOH and N-RGOH at different concentration levels of NO_2_ gas. Results show that N-RGOH revealed a good response at lower NO_2_ concentration. (a-4) Responses of the B- and N-RGOH to 800 ppb NO_2_, 80% RH, 1000 ppm CO_2_, 100 ppm NH_3_, saturated methanol, ethanol, and acetone vapors. Reproduced with permission from [39], copyright 2019 ACS. (**B**) The sensing performances of Pt decorated over highly porous 3D graphene: (b-1) An optical and schematic image of fabricated sensor device. (b-2) SEM image of highly porous 3D graphene; inset shows the TEM image of Pt NP. (b-3) The symmetric response of Pt-3D graphene to various H_2_ concentration levels at 200 °C. Reproduced with permission from [81], copyright 2019 Elsevier.

Wu et al. [39] reported a high-performance NO_2_ sensor at RT using boron (B)- and nitrogen (N)-doped 3D reduced graphene oxide hydrogel (RGOH). Their fabricated sensor displayed significant improvements. The sensing performance details are shown in Figure 6A and are discussed in the introduction section of this article. 

Likewise, Phan et al. [81] reported a fast response and highly sensitive H_2_ sensor using Pt NPs decorated on 3D graphene. Their sensor showed a response value of 16% with response/recovery times of 9/10s to 1% H_2_ concentration at an OT of 200 °C. Moreover, they claimed good stability and linearity towards different gas concentrations. However, they did not report the selectivity of their fabricated sensor (see Figure 6B). 

**Figure 7:**
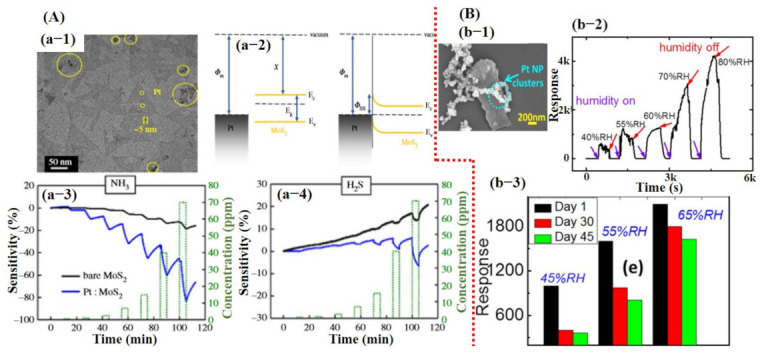
Demonstration of the sensing results for physically and chemically synthesized MoS_2_ sheets: (**A**) Sensing properties of Pt NPs decorated on physically grown MoS_2_ for NH_3_ and H_2_S detection: (a-1) SEM image of monolayer MoS_2_ decorated with Pt NPs. (a-2) The formation of Schottky barrier between Pt–MoS_2_ and barrier height. (a-3,4) Sensor response towards the NH_3_ and H_2_S. Reproduced with permission from [107], copyright 2018 Royal society. (**B**) The sensing performance of Pt NPs decorated on the chemically synthesized MoS_2_ sheets towards humidity: (b-1) SEM image of Pt NPs decorated on MoS_2_. The inset reveals a higher magnified image. (b-2) Sensor response towards different levels of relative humidity. The sensor showed a good response with full recovery, revealing that a chemically synthesized MoS_2_ is a promising candidate. (b-3) Stability test was checked after 1.5 months, and degradation in sensor response was observed. Reproduced with permission from [92], copyright 2018 IOP.

Due to the exceptional performances of 2D graphene/rGO-based sensors, efforts were then made to work on other 2D materials with some band gap, including MoS_2_, MoSe_2_, SnS_2_, VS_2,_ TaS_2_, and WS_2_ for the fabrication of transistor-based devices [6,25,26]. Among them, MoS_2_ was widely studied to explore the sensing performances of TMDCs. At first, transistor devices were made using pure MoS_2_ monolayer and then later on for further enhancement in sensing performance hybridization of the MoS_2_, with metal/metal oxide catalyst being investigated. For instance, the sensors made of pure MoS_2_ showed little response towards different gases but were not able to display full recovery and good selectivity [106]. Park et al. [107] reported enhanced NH_3_ and H_2_S detection using Pt NPs decorated on MoS_2_-synthesized via vapor deposition technique. Their sensor revealed relatively better performance than that of pure MoS_2_ sensors but still did not achieve full recovery to the baseline (see Figure 7A). Moreover, they did not report cross-sensitivity with other gases. MoS_2_ synthesized via physical route did not reveal good performance [106,107]. Therefore, chemically synthesized MoS_2_ sheets were investigated by Burman et al. [92]. They reported Pt NPs decorated on chemically synthesized MoS_2_ flakes for humidity sensing. Fabricated sensor loaded with 25% Pt NPs showed a maximum response. The sensors demonstrated excellent response towards different percentages of humidity with full recovery to baseline, but they too did not report selectivity of their sensors. To check the long-term stability, they tested the sensor after 1.5 months, and a clear degradation in response was observed indicating the highly unstable nature of the synthesized material [92]. The sensing results for the fabricated sensor are summarized in Figure 7B. 

Besides carbon materials and TMDCs, metal oxide was also broadly investigated through various techniques, such as hybridization, p–n junction formation, and core–shell structures [27,28,37,50,108]. As discussed in the computational section, oxygen vacancies are vital for metal oxide-based sensors. The type and number of oxygen vacancies depend upon the OT (O_2_^−^, O^−^, and O^2−^). Usually, sensors perform well at high temperatures due to the higher number of oxygen vacancies and higher reaction rate between the target molecule and sensing material surface [45]. Likewise, for carbon and TMDCs sensors, the decoration of metal catalysts was needed to further improve the sensor performance together with selectivity. 

**Figure 8:**
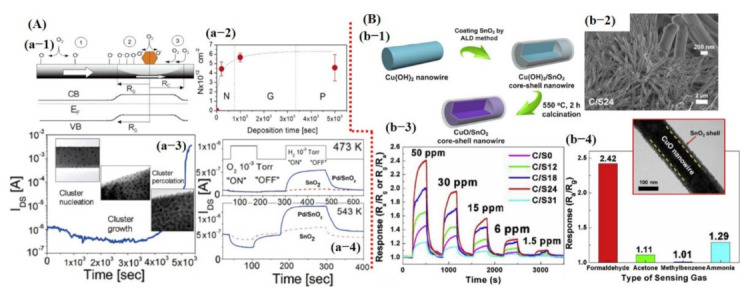
Metal oxide-based gas sensors: (**A**) Pd NPs decorated on the single SnO_2_ NW for O_2_ and H_2_ detection: (a-1) The formation of the depletion region on the nanowire (NW) surface with the Pt NP deposition. As shown in Figure 8 (a-1), the depletion region facilitated in increasing the oxygen adsorbent on the sensing material and consequently improved the sensor performance. (a-2) The Pt NP deposition rate. (b-3) IDS was measured during Au deposition. The onset of the large current increased beyond ≈4 × 10^3^ s can be attributed to the percolated pathways generated through excessive Au particles. (a-4) The sensing performances of pure SnO_2_ and Pd–SnO_2_ NW to O_2_ and H_2_. Reproduced with permission from [109], copyright 2005 ACS. (**B**) Sensing performance of CuO@SnO_2_ core–shell structure for formaldehyde detection. (b-1) Schematic diagram of the fabrication process. (b-2) SEM image of core–shell NW structure. (b-3) Sensor response with different shell thickness; SnO_2_ with 24 nm thick shell revealed maximum response. (b-4) Selectivity graph. The inset revealed the TEM image of the core–shell NW structure. Reproduced with permission from [111], copyright 2019 Elsevier.

Kolmakov et al. [109] fabricated sensors using Pd catalyst decorated on a single SnO_2_ NW. They indicated enhanced sensing response to oxygen and H_2_ gases. To understand the catalytic effect of Pd, they observed I_DS_ current value during Pd deposition. At the start, a reduction in I_DS_ value was noted, which indicated that the Pd NPs on the NW surface created Schottky barrier-type junctions resulting in the formation of electron depletion regions within the NW. However, at certain Pd deposition times, a dramatic increase in conductance was observed, representing percolation among the NPs, eventually leading to shorting of the SnO_2_ NW. They explained that the formation of depletion zones through the Schottky barrier plays a vital role to enhance the sensor performance by increasing the population of oxygen vacancies in that particular region [109] (see Figure 8A). Therefore, considerable research is being devoted to attaining the depletion zone through the Schottky barrier, p–n junction formation, layer by layer, and core–shell NSs [34,48,50,52,58,109,110]. 

Recently, Zhu et al. [111] fabricated hetero-structured p-CuO/n-SnO_2_ core–shell NWs by combining a solution and atomic layer deposition process for enhanced sensitive and selective formaldehyde detection. They indicated that SnO_2_ shell thickness is crucial; 24 nm thick SnO_2_ shell showed a high sensitivity of 2.42, a threefold higher response than that of pristine CuO NW towards 50 ppm formaldehyde (HCHO) at 250 °C. The enhanced gas sensing performance could be attributed to the formation of p–n heterojunction through specific band alignment and the heterojunction-depletion model. The cross-sensitivity was also checked with three other gases/compounds; NH_3_ showed a comparatively higher response of 1.29 than that of acetone and methylbenzene [111] (see Figure 8B).

**Figure 9:**
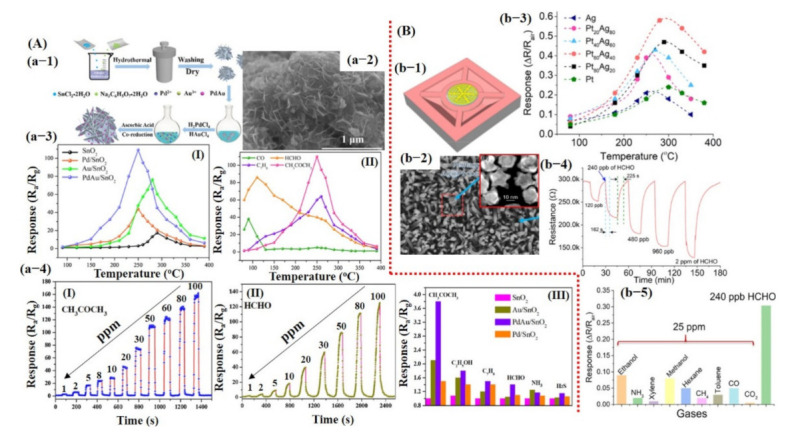
Metal oxide-based gas sensors: (**A**) Pd/Au NPs decorated on the SnO_2_ nanosheets for temperature-dependent acetone and HCHO detection: (a-1) Synthesis process of Pd/Au NPs decorated on the SnO_2_ nanosheets. (a-2) The SEM image for the Pd/Au-decorated SnO_2_ nanosheets. (a-3) The temperature-dependent response of the as-fabricated sensors: (I) sensing performance of SnO_2_, Pd@SnO_2_, Au@SnO_2_, and Pd/Au@SnO_2_ to 50 ppm acetone, with Pd/Au@SnO_2_ demonstrating maximum response at 250 °C; (II) Pd/Au@SnO_2_ sensor response towards various compounds (50 ppm) at different OT, showing maximum response for HCHO @ 110 °C and acetone @ 250 °C. (a-4 (I, II)) The sensor response @ 250 °C and 110 °C for a wide range of acetone and HCHO concentration levels, respectively. (a-4 (III)) The responses toward 1 ppm acetone compared to 1 ppm of the other common interfered biomarker gases. Reproduced with permission from [57], copyright 2019 Elsevier. (**B**) Ag@Pt core–shell NSs on the ZnO NWs for HCHO detection: (b-1) Fabricated chemiresistive sensor device. (b-2) SEM image of Ag/Pt core–shell on ZnO NWs. The inset reveals the magnified image of Ag@Pt core–shell NSs. (b-3) Sensor response by varying the Pt and Ag content ratio. The Pt60 and Ag40 showed a maximum response at 280 °C and were selected for further measurements. (b-4) Optimized sensor response toward different concentration levels of HCHO. (b-5) Sensor response to other compounds. Reproduced with permission from [58], copyright 2020 ACS.

Similarly, Li et al. [57] reported a Pd–Au nanoparticle (NPs) decorated on SnO_2_ nanosheets (NShs) for temperature-dependent acetone and formaldehyde detection. Xu et al. [58] also reported Ag@Pt core−shell nanostructures (NSs) decorated on ZnO nanowires (NWs) for formaldehyde detection. Their results are shown in Figure 9A,B and discussed in the introduction section of this article.

### 2.2. Current Challenges in Chemical Gas Sensors

From the aforementioned state of art survey, one can see that the explored materials and techniques can certainly assist in achieving the milestone for maturation of the sensor industry. These techniques significantly enhance the sensing properties in terms of sensitivity, response/recovery time, repeatability, the limit of detection (LOD), and OT. However, excellent selectivity with long-term stability is still a great challenge. Materials tend to lose their properties with the environmental effects and aging, thereby causing degradation in the sensor response, which is known as the drift error. Most of the studies did not consider the aging effect (long-term stability) which is essential for sensor implementation in a real-world application. On the contrary, great efforts have been devoted to developing a sensing material for highly selective detection of a particular gas/compound. The reported sensors demonstrated higher sensitivity towards a specific gas/compound; however, were not able to completely neglect the other gases and showed cross-sensitivity with small response values. 

Additionally, the metal NSs, which are considered and reported as good catalysts for a particular gas, have been used for the selective detection of various other gases, showing significant improvement in selectivity. For instance, Pt is considered the best catalyst for H_2_ sensing but it is also used for other gases/compounds and has shown competitive results. Pt NPs decorated on physically grown WO_3_ NRs showed high sensitivity towards H_2_ gas [112,113]. Pt NPs of a similar material decorated on physically synthesized WO_3_ hemitube [114] demonstrated a good response to H_2_S and acetone, suggesting in-depth investigation for proper understanding of NSs effect on sensing even with same material synthesized in similar manners. Pt decorated on WO_3_ synthesized via the same physical route showed promising sensing performance for NO [115] and SO_2_ sensing [116]. Similarly, Au-doped ZnO gas sensors showed a good response to a number of gases including NH_3_ [117], CO [118], and HCHO [110]. Recently, nanosized Au loaded on ZnO NPs was developed for toluene detection, achieving ultrasensitive output performances [48]. Furthermore, Ni-doped ZnO demonstrated a good sensing response towards NH_3_ [119] and HCHO [120], leaving a big question on the selectivity. Therefore, in-depth analysis and investigation are needed to apprehend the surface charge distribution on various facets of sensing material nanostructures (nanoparticles, nanopyramid, nanoporous, nanocubes, nanowires, nanorods, nanotubes, etc.) through computational (to find a site/facet that allows maximum adsorption and charge transfer between target molecules and surface) and experimental studies. 

In parallel with the development of novel material, it is equally indispensable to find facile, efficient, and smart alternatives to improve the selectivity and to compensate for the drift error without compromising other sensor performances

## 3. Machine Learning-Based Smart Gas Sensors

**Figure 10:**
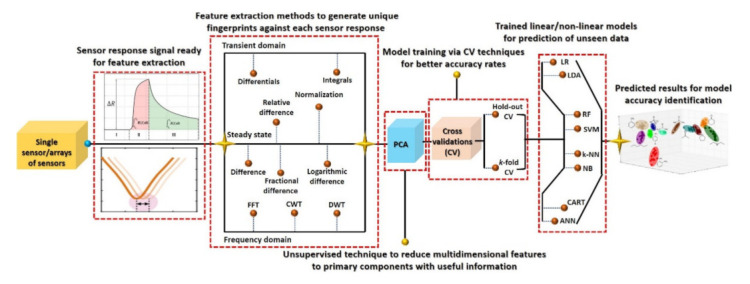
Overall machine learning process from sensors response, data processing, and model training to prediction accuracy.

**Figure 11:**
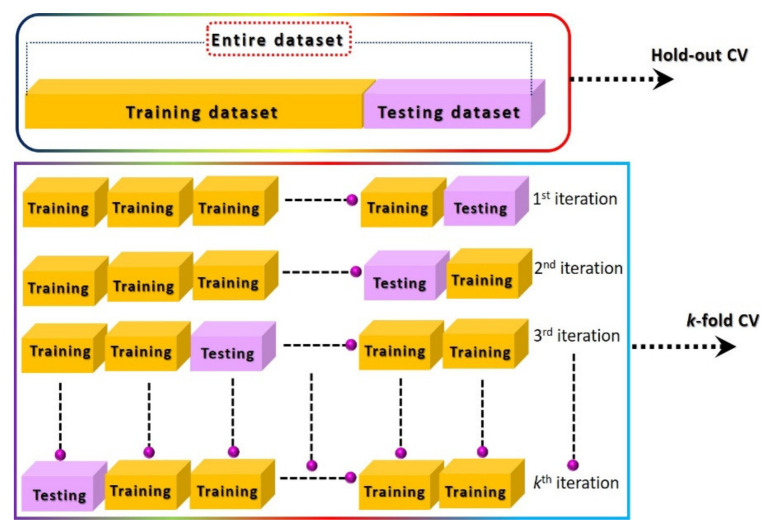
Illustration of hold-out and *k*-fold cross-validation techniques.

Machine learning has emerged as a potential data-processing approach to improve selectivity and to compensate for drift error. However, it requires a large amount of labeled data under different circumstances for accurate training of classifier models [59,66,121]. A large dataset (with hundreds/thousands of examples) of a sensor response to a particular analyte will probably result in high redundancies due to the co-existence of data points. Therefore, a careful selection and gradation of important features are highly recommended using variance analysis test (ANOVA F-test) and heat maps in order to avoid the data overlapping. The obtained important feature vectors are used as an input to the PCA for the generation of a unique signature marker for a specific analyte. Although PCA further reduces the dimensionally, all the information from the input features are efficiently retained by projecting the data in reference space with best fit linear slop. The feature variance across the linear fit curve is the obtained first principal component of the input data. Figure 10 illustrates the overall machine learning process including feature extractions through various data-processing techniques and different linear/nonlinear models and their training methods for accurate prediction. As indicated, initially it is essential to identify the useful features from the dynamic response curves (steady-state, transient, and frequency domain), process them using an unsupervised PCA algorithm for dimensionality reduction, and visualize the unique signature pattern against each class. Afterward, the models are trained on the obtained signature patterns through appropriate cross-validation (CV) approaches for the realization of the best fit model with maximum accuracy. The final step is the accuracy testing of the trained models on the unknown dataset [122,123,124,125,126]. Various kinds of features and their extraction techniques for chemiresistive and FET sensor devices are discussed in the following sections. Principal component analysis (PCA) is one of the most widely used unsupervised techniques in the literature. The main contribution of this technique is to generate unique fingerprints and reduce the computational power during model training by eliminating the redundancy of a big dataset through the diagonalization of the correlation matrix. Appendix A presents basic information on PCA calculations. During model training, the optimization of the model algorithms through different CV methods is essential to avoid overfitting and to acquire maximum and stable prediction accuracy rates. The CV is a statistical method used to approximate the accuracy of a machine learning model. The hold-out and *k*-fold (stratified *k*-fold CV, leave-one-out (LOOCV), leave p-out (LPOCV), etc.) are the commonly reported CV techniques [122,125,126]. The selection of an appropriate CV technique for a particular dataset is the key to develop a highly efficient machine learning model. The hold-out is one of the most basic and simple approaches in which the entire dataset is first randomly shuffled and then divided into two datasets: training and testing (for training and validation of model accuracy, respectively), as shown in Figure 11. Mostly, the training dataset (60–80%) ratio is set higher than the testing dataset (40–20%) in order to increase the model training probability rate on all available examples in the dataset. However, random shuffling of the dataset may alter the whole training process and thus reveal unstable prediction accuracy. Additionally, it is not suitable for a small amount of datasets since it randomly shuffles the entirety of the data into two subsets (training and testing), and it is highly likely that the model may not be trained on all existing examples in the dataset. As a result, it may lose accuracy. The hold-out approach is considered more suitable for a large dataset in which the possibility of model training on all examples will be much higher with relatively lower computational power requirement during model training. On the contrary, the *k*-fold CV is considered a promising approach for comparatively smaller datasets and to address the hold-out limitations. In this method, the entire dataset is split into *k* subsets, in which every data point will take part in training and testing of the model, as shown in Figure 11. To achieve the training and testing at each data subset, the machine learning algorithm runs for *k* iterations (Figure 11). The accuracy of the model is calculated at each iteration and then averaged after the *k*th iteration to attain the final accuracy rate of the model. Since this CV method trains and tests the model at each data point, the probability rate for training on each example will be much higher, even with small datasets. The selection of *k* value is highly sensitive to the nature of the input dataset for accurate training of a model. The implementation, advantages, and accuracy of these techniques for various chemiresistive and FET sensors/e-nose systems are covered in the next section. 

**Figure 12:**
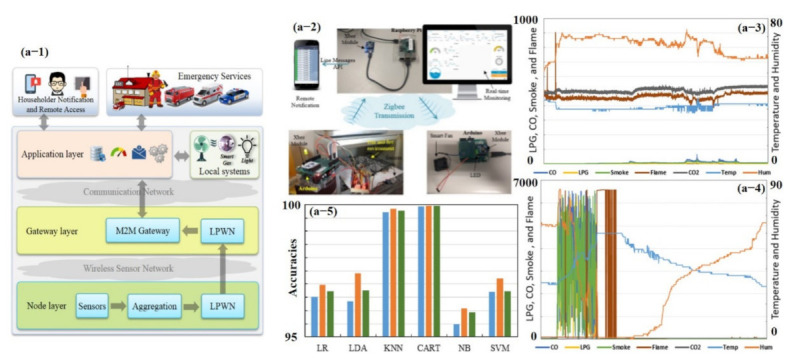
Early fire detection in smart homes using machine learning: (a-1) overall system overview, (a-2) whole hardware and experimental setup, (a-3) sensor measurements taken under usual conditions, (a-4) sensor measurements taken under extreme conditions, and (a-5) prediction accuracy histogram before and after replacing the missing values with the mean values computed from the training data. Reproduced with permission from [73], copyright 2020 IEEE.

In the past few decades, numerous studies have been conducted on electronic nose (e-nose) systems, which consist of an array of different gas sensors that interact with a broad range of chemicals of varying strengths, feature extraction, and pattern recognition algorithms that process and extract useful information to generate unique fingerprints [70,127,128]. Recently, Salhi et al. [73] developed a whole hardware setup (from sensing to training and testing of different classifiers) for early gas leakage detection in smart homes (see Figure 12). They divided the setup into three logical layers: node layer, gateway layer, and application layer. The node layer mission is to collect and process the data, then transmit it through a low-power wireless network (LPWN) such as ZigBee, Z-Wave, and Bluetooth devices to the gateway layer. The gateway layer collects the data from the node layer and can be accessed remotely by the end-user as a control and monitoring system. Data are then examined by the application layer. This layer serves as the interface between machine to machine (M2M) home network and M2M devices. The main purpose of this layer is to evaluate and correlate data received from the gateway layer to detect anomalous patterns; for instance, predicting gas leakage and fire incidences in a smart home environment [73]. Their e-nose consists of seven commercial sensors, including temperature, humidity, LPG, CO, CO_2_, smoke, and flame. Data were collected under usual and extreme conditions during 1.5 days, and 21,146 measurement samples were obtained. Each sample counts seven values, i.e., one value for each sensor. Every sample is collected periodically every 5 s. In the study, six classifiers were trained using 10-fold cross-validation (CV) with the existing dataset (training 80% and testing 20%): logistic regression (LR), linear discriminant analysis (LDA), *k*-nearest neighbors (KNN), classification, regression trees (CART), Gaussian naïve Bayes (NB), and support vector machines (SVM). Their results revealed the largest estimated score using CART (99.93%) and with KNN (99.71%) accuracy for the given dataset. They further improved the accuracy by computing the mean values in the training dataset and replacing them with missing data [73]. Though they were able to develop very selective smart sensors with almost 100% accuracy, their setup was very large, relatively costly, and consumed more power [73]. The overall setup collected data, and the result is shown in Figure 12.

To reduce the power consumption and to develop an economical setup, researchers fabricated and reported single-sensors based on selective material synthesized through earlier mentioned techniques [124,129]. These sensors generate distinct dynamic responses against different gases, and hence one sensor can be used for the identification of various gases. The details are discussed in the coming sections.

### 3.1. Chemiresistive Type Smart Gas Sensors Using Machine Learning

**Figure 13:**
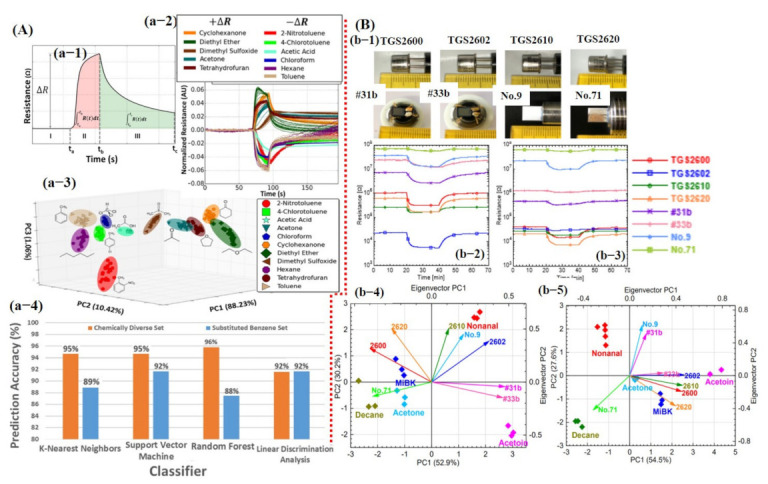
Smart sensing using machine learning: (**A**) Sensing of several compounds using single graphene-based chemiresistive sensor: (a-1) Typical response of chemiresistive sensor, regions I, baseline; II response time; and III, recovery. It indicates the potential features that can be extracted including the maximum change in resistance (ΔR), area of the response (red shaded area), and area of the recovery (green shaded area). (a-2) Normalized response for all compounds, ready for feature extraction. (a-3) PCA scores for the 11 compounds revealed small overlapping. (a-4) Different classifier prediction accuracy histogram, reproduced with permission from [123], copyright 2016 ACS. (**B**) e-nose system for smart detection of different compounds under dry and humid air: (b-1) Optical image for all the sensors. (b-2,3) Sensor response under dry and humid air, respectively. No. 9 and no. 71 sensors were not affected by humidity. (b-4,5) Corresponding PCA results of all sensor data in air and humid environment; PCA also confirmed the little humidity effect on no. 9 and no. 71 sensors. Reproduced with permission from [130], copyright 2017 MDPI.

Enormous efforts have been made to improve the sensing ability of chemiresistive-type sensors using various machine learning techniques. A typical resistive type gas sensor shows a change in resistance upon exposure of gas molecules. An increase/decrease in resistance depends on the nature of the sensing material and target molecule [42,43]. An output dynamic response signal with appropriate labeling of baseline, response time, and recovery time is shown in Figure 13Aa-1. Performance enhancement of a smart sensor mainly depends on three factors: (1) use of appropriate sensing materials, (2) useful feature extraction and data processing techniques, and (3) efficient training of single model/multiple models. It has been reported that model/classifier accuracy can be significantly improved using cascading of multiple classifiers. For example, Guney et al. [122] reported classification of n-butanol concentrations using k-NN and SVM. The decision tree structure was used to extract the features, and then K-NN and SVM classifiers were trained on these features using the leave one out (LOOCV) technique. The LOOCV method is a type of k-fold CV where k = N and N is the total number of the data points. This method certainly helps to improve the accuracy rate but it is recommended for small datasets as the number of iterations is equal to the number of data points. Thus, for bigger datasets, it will require higher computational power and time to train a model. The K-NN (93%) and SVM (96%) classifiers with decision tree models showed great improvement in accuracy compared with singular SVM (86%) and K-NN (87%) classifiers [122]. 

For one to obtain a PCA graph with great discriminating ability among different classes, a careful selection and gradation of important features is highly recommended using a variance analysis test (ANOVA F-test) and heat maps in order to avoid the data overlapping. For example, Faleh et al. [131] studied the recognition of ozone (O_3_) using an array of four WO_3_ sensors and PCA calculations. They reported that the static parameter R_gas_/R_air_ is not sufficient to distinguish among various concentrations of the target gas. Therefore, for better discrimination among various concentrations, they used the area under the response time curve from the dynamic (transient) response. It was concluded that using the response time parameter, the class separation among different concentrations was much better than the resistance ratio [131]. Later, Nallon et al. [123] used unmodified graphene as a single sensor for discrimination among different chemicals/compounds. Their fabricated sensor was successfully able to differentiate among 11 different compounds without any considerable overlapping. Excellent PCA results can be attributed to the features selected with the most useful information. For each measurement (11 compounds, 20 repetitions), ΔR, A_Resp_, A_Recov_, and A_Resp_/A_Recov_ features were calculated to create a 4 × 220 feature vector as an input to the PCA generator. Each row represents a single measurement and each column represents a feature calculated for that particular measurement. To avoid overfitting, the dataset was divided into 60% training and 40% testing size. The above-mentioned feature vectors were used as input to train the KNN, linear SVM, RF, and LDA classifiers using 10-fold CV for accuracy comparison. The overall classification accuracy for 11 compounds was noted above 90% for every classifier with an accuracy rate of 95%, 95%, 96%, and 92% for KNN, SVM, RF, and LDA, respectively (see Figure 13A). Additionally, most encountered misclassifications were analyzed through the confusion matrix [123]. Itoh et al. [130] reported highly selective VOC detection using different kinds of sensors in humid and pure air environments. The sensing array was composed of four commercially available semiconductor metal oxide sensors (TGS 2600, 2602, 2610, and 2620; Figaro Engineering Inc., Minoh, Japan); two semiconductor Pt, Pd, and Au/SnO_2_ sensors; and two semiconductor Zr-doped CeO_2_ sensors (bulk-type sensors). Response from all eight sensors to 1 ppm of acetone was collected under a dry and humid air environment. It was found that bulk type sensors were not affected by the humidity ascribed to the different sensing mechanisms (see Figure 13B). The PCA results were obtained using response values from the sensors. First, normalized scores ($x_{ti}$) were calculated using Equation (1) [130]:

(1) $$x_{ti}=\frac{\left(r_{ti}-\bar{r_{t}}\right)}{\sigma_{t}}$$

where t is the sensor index, i is the sensor response analysis index, $r_{ti}$ is the sensor response analysis i of sensor t, $\bar{r_{t}}$ is the average sensor response of sensor t, and $\sigma_{t}$ is the standard deviation of sensor t.

(2) $$\left|A\right.-\left.\lambda E\right|=0$$

where A is the matrix displayed in Equation (3) [130], λ is the eigenvalue, and E is the unit matrix.

(3) $$A=\left(\begin{array}{c} \begin{array}{cccc} C_{11} & C_{12} & \cdots & C_{1m}\\ C_{21} & C_{22} & \cdots & C_{2m}\\ \vdots & \vdots & \ddots & \vdots \\ C_{m1} & C_{m2} & \cdots & C_{mm} \end{array} \end{array}\right)$$

where$\;C_{ab}$ is a correlation coefficient between sensors a and b $(C_{11}=$ $C_{22}=\cdots =C_{mm}=1)\;\mathrm{and}\;m$ is the maximum number of sensors. The eigenvalues can be acquired for each sensor index, as shown in Equation (4) [130].

(4) $$\lambda =\lambda_{1},\;\lambda_{2},\;\ldots \lambda_{m}\;(\lambda_{1}>\lambda_{2}>\ldots \;\lambda_{m}\;)$$

where$\;C_{ab}$ is a correlation coefficient between sensors a and b $(C_{11}=$ $C_{22}=\cdots =C_{mm}=1),\;\mathrm{and}\;m$ is the maximum number of sensors. The eigenvalues can be acquired for each sensor index, as shown in Equation (4).

(5) $$AV_{j}=\lambda_{j}V_{j}\;\ldots \ldots \;Or\;\left(A-\lambda_{j}E\right)V_{j}=0\;\left(\begin{array}{c} v_{1j}\\ v_{2j}\\ \vdots \\ v_{mj} \end{array}\right)$$

Finally, the PCA $\left(Z_{ji}\right)$ was calculated by the product sum of the normalized score and eigenvectors, as shown in Equation (6) [130]:

(6) $$Z_{ji}=v_{1j}x_{1i}+v_{2j}x_{2i}+\cdots +v_{mj}x_{mi}$$

Figure 13B shows the calculated PCA scores, confirming that bulk type sensors were not affected by the humidity. The PCA was performed for class discrimination. Classifiers were not trained for accuracy checks [130].

**Figure 14:**
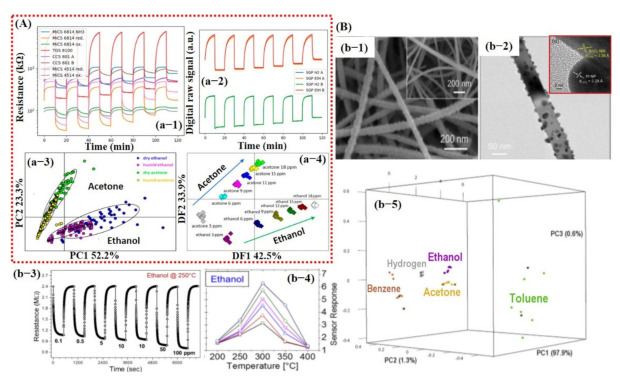
(**A**) e-nose system consisting of analog and digital sensors for detection of different compounds: (a-1) Raw data of analog sensors to acetone under humid conditions. (a-2) Raw data of digital sensors after exposure to acetone under humid conditions. (a-3) PCA score plot showing the effect of humidity. The measurements are labeled by condition and target compound. (a-4) LDA scores plot for the discrimination of compounds and concentrations of the measurements performed under dry and humid conditions. Reproduced with permission from [132], copyright 2019 ACS. (**B**) Smart sensing of different compounds using thermal fingerprints of a single sensor based on Pt NPs@SnO_2_ NWs. (b-1) SEM image of Pt NPs decorated on SnO_2_ NWs. (b-2) Higher magnified TEM image of single SnO_2_ NW decorated with Pt NPs. (b-3) Dynamic response during the exposure of different ethanol concentrations @ 250 °C. (b-4) Sensor response to ethanol at different operating temperatures. (b-5) Three-dimensional plot of the principal components revealing excellent discrimination among different compounds. Reproduced with permission from [133], copyright 2019 Elsevier.

Jaeschke et al. [132] demonstrated an innovative e-nose system using a unique combination of analog and digital MOx sensors for ethanol and acetone detection in dry and humid environments. The sensing array consisted of 8 analog and 10 digital sensors. For PCA graphs, features and feature extraction methods were not described in their article. Nevertheless, they showed a good PCA graph. The entire dataset was divided into 75% (300 measurements) for training and the remaining 25% for testing (102 measurements). The hold-out CV method was used to train the LDA model. The maximum 76.4% LDA accuracy was recorded for the classification of different VOC concentrations [132] (see Figure 14A). The lower accuracy rate might have been due to the inappropriate selection of the CV method since the hold-out method randomly shuffled the entire dataset for training and testing. Therefore, the probability of model training on all kinds of data examples might have been very low and may have caused the degradation in the model performance.

In the same year, Tonezzer et al. [133] reported a single sensor (Pt-decorated SnO_2_ nanowires and pure SnO_2_ NWs [134]) that is highly selective using thermal fingerprints. They checked the response of five different gasses, namely, ethanol, acetone, benzene, toluene, and H_2_, at different temperatures. The sensor response checked at different temperatures was then used as thermal fingerprints for feature extraction. With only one nanostructured material (Pt–SnO_2_) and five temperature values, their system was able to qualitatively and quantitatively discriminate all the gases with high accuracy [133] (see Figure 14B). The entire thermal fingerprint data were divided into 70% training (175 measurements) and 30% testing (75 measurements) subsets and then were used to train the SVM model. Their trained model was able to differentiate among seven different gases and chemicals with an accuracy rate of 100%. This was attributed to the selection of thermal fingerprints as feature input vectors for model training. However, the collection of input feature vectors at different operating temperatures for each gas/chemical may not be very feasible for sensor application in the real environment.

**Figure 15:**
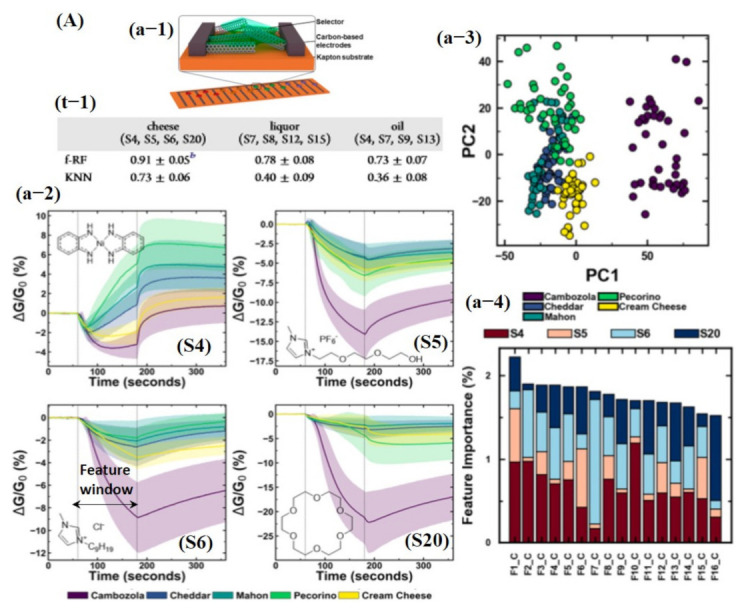
(**A**) Food classification using an array of 20 chemiresistive sensors and machine learning: (a-1) Schematic diagram of functionalized carbon nanotube (CNT) chemiresistive device. (a-2) Sensing response for S4, S5, S6, and S20 toward five kinds of cheeses. The response is represented as a change in conductance normalized to the conductance at the start of the exposure (ΔG/G0). Each exposure started at t = 60 s and ends at t = 180 s (marked by dashed vertical lines used as features for KNN training). Each response was an average of 40 separate sensing experiments. The shaded area represents the standard deviation of the response. (a-3) PCA of extracted features from the five-cheese dataset showing the first two principal components. (a-4) Top 16 overall most important features in the five-cheese, wherein (t-1) demonstrates the prediction accuracy toward different kinds of cheese using KNN and f-RF classifiers. Reproduced with permission from [135], copyright 2019 ACS.

**Figure 16:**
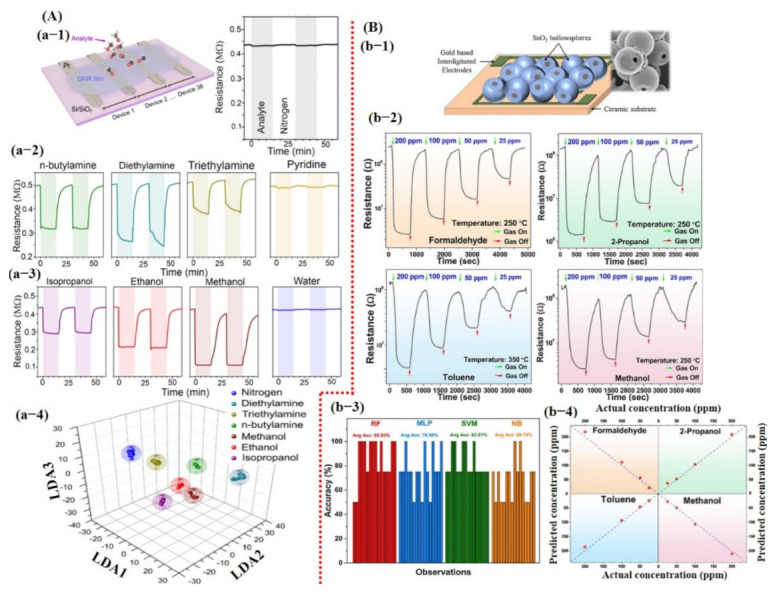
(**A**,**B**) summarize the detection results for different VOCs using chemiresistive devices and machine learning. (a-1) Schematic diagram of graphene nanoribbon sensors. (a-2) Real-time resistance change response toward different amines and alcohols. (a-3) 3D LDA graph representation with 100% accuracy. Reproduced with permission from [129], copyright 2020 ACS. (b-1) Schematic diagram of fabricated hollow SnO_2_ sphere-based chemical sensor; inset presents the SEM image of synthesized hollow spheres. (b-2) Real-time resistance change response to different chemicals at various concentration levels. (b-3) Average accuracy bar graph for each model. (b-4) Representation of concentration prediction for each chemical. Reproduced with permission from [126], copyright 2020 Elsevier.

Besides chemicals and gas detection, chemiresistive-based sensors were also used to detect the quality of food using machine learning. For instance, Schroeder et al. [135] recently fabricated a chemiresistive sensing array using 20 different functionalized CNT sensors for classification of food quality using machine learning [135]. In order for one to build a sensing system that can differentiate between complex organic odor mixtures, the choice of sensors is critical. Various types of the functional groups were attached on the surface of CNTs, including transition metal complexes (S1, S2, S3, and S4) to bind organic acids and sulfur-containing compounds; ionic liquids (S5, S6, S7, and S8) to interact with ketones, aldehydes, alkanes, and aromatic compounds; porous polymers (S9, S10, S11, and S12) to detect a large number of organic vapors; cavitand and molecules (S13, S14, S15, S16, and S20) for detection of aromatic compounds and alcohols with size-exclusion properties; and metalloporphyrins (S17 and S18) to bind amines, alcohols, ketones, alkanes, and aromatic compounds [135]. For data processing to train a KNN model, features were directly collected from a specific window (120 s of exposure and 180 s of recovery) of the dynamic response, highlighted with a dotted line in Figure 15Aa-2. Later, tsfresh was also used to extract the features from the dynamic response, and 794 features were extracted, ranging from the coefficients of a CWT or FFT to parameters such as time series length, mean, max, and median, as well as many others. Featured random forest (f-RF) models were built on the tsfresh-extracted features, while the KNN models used the raw time series data (un-normalized) with the nearest neighbor of 1 supplying the class vote. The dataset was split into 80% training and 20% testing subsets. The f-RF (featured trained random forest) model was trained with 50 iterations, and after each iteration, the data were randomly shuffled. The accuracies from all 50 iterations were then averaged, and a standard deviation was calculated to determine overall model accuracy. The accuracies were calculated, and it was found that the f-RF model displayed maximum accuracy of ≈91% in predicting cheese with a specific set of sensor data (S4, S5, S6, and S20) (see Figure 15A). As expected from the dynamic response, feature-extracted PCA scores discriminated cambozola cheese, but some overlapping among other kinds of cheese was found. Moreover, the status of the tsfresh extracted was also checked, and 16 features were found with more useful information to accurately train the f-RF model (see Figure 15A). Among these 16 features, CWT and FFT were the most commonly occurring features. Therefore, it can be estimated that CWT and FFT are vital and contain much of the useful information for unique pattern generation [135]. Shekhar et al. [129] reported a CVD-grown graphene nanoribbon e-nose system consisting of 38 sensors for VOC detection. The schematic diagram, output response, and LDA accuracy graphs towards different VOCs are shown in Figure 16A. All 38 sensors showed the strongest response towards amines and alcohols. Therefore, amines and alcohol response datasets were used for model training. The response curves of various amines and alcohols were collected and normalized using Equations (7) and (8) [129] for their implementation as a feature vector to train an LDA model.

(7) $$N=\frac{S_{i}}{S_{avg}}$$

(8) $$S_{avg}=\frac{\sum_{k=1}^{n}S_{k}}{n}$$

where N is normalized data for model training, $S_{i}$ is response of $i^{th}$ sensor, $S_{avg}$ is the average response of all the sensors, and n is the number of sensors. The LDA model trained on the normalized dataset showed 100% accuracy. However, the details on model training were not discussed. Recently, Acharyya et al. [126] presented VOC detection using single SnO_2_ hollow sphere sensors and machine learning. The sensor schematic diagram, response, and accuracy of different models are summarized in Figure 16B. The sensor responses at different operating temperatures (varying from 200 to 350 °C), VOC concentration, response, response time, and recovery time were used as input features for model training. Different classifiers were studied, including RF, MLP, SVM, and NB, in order to develop the best fit model for the available input dataset. On the basis of these input feature values, the researchers allowed the algorithms to run using the 16-fold CV technique in order to examine the accuracy for the different test datasets. Then, the results were averaged to produce final accuracy, which is defined as the ratio of the number of correct predictions to the total number of samples. The maximum accuracy was obtained around 85.93% for the RF model. The comparison in Table 1 shows various chemiresistive sensors/e-nose systems with a variety of extracted features and output accurate results.

### 3.2. Field Effect Transistor-Based Smart Gas Sensors Using Machine Learning

**Figure 17:**
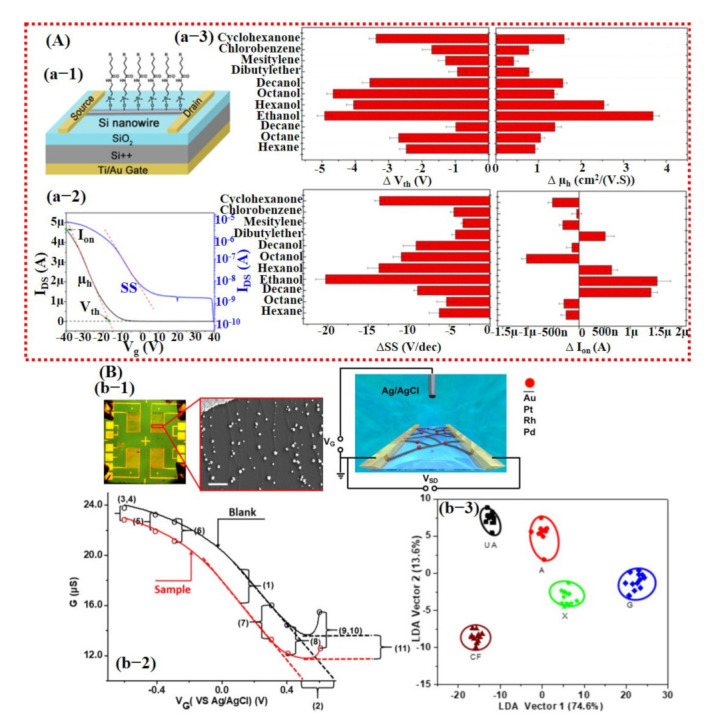
Smart gas sensing using FET-based devices and machine learning. (**A**) Modified single Si NW-based FET device for VOCs detection: (a-1) Schematic diagram of the fabricated FET device. (a-2) Typical IDS response curve of FET device labeled with the extracted features (V_th_), ion, hole mobility, and SS for data processing. (a-3) Variations in (a) V_th_, (b) μ_h_, (c) SS, and (d) ion of sensor S4 upon exposure to various VOCs at a concentration of pa/po = 0.08. Reproduced with permission from [137], copyright 2014 ACS. (**B**) Metal catalyst decorated on the CNT-based FET device for detection of purine compounds: (b-1) Optical and schematic images of the fabricated device. The inset reveals the SEM image of CNTs decorated with NPs. (b-2) Pt NP-decorated NTFET response with and without caffeine (1 mM) solutions. Selected features (11) were calculated from the NTFET curves before and after exposure of caffeine solutions. (b-3) LDA plots for purine compound discrimination. Reproduced with permission from [139], copyright 2019 ACS.

Field-effect transistors (FETs) have also been widely studied for gas/chemical sensing due to their small size and trace level molecule detection with high sensitivity. A FET device consists of three terminals: drain (D), source (S), and gate (G). The current flows from the drain to the source (I_DS_) can be controlled by applying a voltage load at the gate [136]. The sensing materials of FETs are usually based on thin films or monolayers (graphene, TMDCs, and semiconductors) functionalized/decorated with ligands and catalysts [136]. In comparison with resistive type sensors, they are more complex and expensive to fabricate. A typical FET sensing device shows a change in the I_DS_ curve upon exposure to a target molecule within a particular range of gate voltage (V_g_). Similar to resistive-type sensors, selective sensing material, useful feature extraction, and proper model training is vital to developing a smart FET sensor with the ability to accurately discriminate among various gases. In 2014, Wang et al. [137] reported functionalized single silicon (Si) nanowire (NW) for accurate detection of 11 VOCs using artificial neural networks (ANN). Features were extracted from the original and logarithmic I_DS_ curve after gas exposure, with V_g_ ranging from 40 to −40 V. The threshold voltage (V_th_), hole mobility, (µ_h_), and I_on_ (defined as I_DS_ @ V_g_ = −40 V) were obtained and calculated from the original I_DS_ curve, while subthreshold swing (SS) was acquired from logarithmic IDS curve and was used as input features for a neural network. A response curve is shown in Figure 17A, which is labeled with extracted features and the variation graphs for the features upon exposure to different VOCs. Their trained ANN was perfectly able to recognize 11 VOCs and their binary/ternary combinations as well [137]. Similarly, Guo et al. [138] developed an ANN flexible gas sensor based on ultra-large area MoSe_2_ nanosheet. They proposed a machine learning and data-driven approach to predict the location of the gas source at home in a macroscopic scene. The process consisted of four layers, an input layer, two hidden layers, and an output layer. The input layer is the data array acquired from the sensors, while the output layer is the location of the gas source. Their proposed approach achieved a satisfactory prediction accuracy for NO_2_ and NH_3_ [138].

In 2019, Bian et al. [139] synthesized a sensing array using different metal catalysts decorated on single-walled carbon nanotube (SWCNTs) to develop a FET device for the detection of purine compounds (adenine, guanine, xanthine, uric acid, and caffeine). The 11 different features were extracted from the response curve of the FET device. The response curve labeled with extracted features is shown in Figure 17Bb-2. For training, the entire dataset was split into 10 subsets, with each subset containing the ratio of caffeine to non-caffeine of 1:2.54. Nine subsets were used to build the model, and one subset was used for testing the robustness of the model. The stratified 10-fold cross-validation was used to train the SVM model. The stratified k-fold CV is an efficient approach to shuffle the entire dataset and then divide it into equal subsets with a good representation of all the training examples. Therefore, their trained SVM model was successfully able to distinguish caffeine with an accuracy rate of 93.4% [139] (see Figure 17Bb-3). 

**Figure 18:**
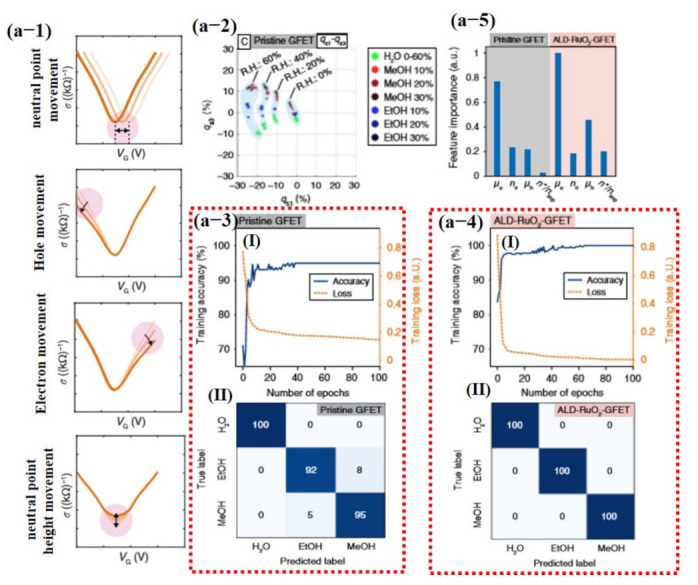
Pristine graphene and AL-RUO_2_-based GFET devices for detection of different compounds under dry and humid environments. (a-1) Schematic illustration of extracted features for data processing, movement of the charge-neutral point (Np), variation in hole branch, variation in electron branch, and variation in height of the charge Np. (a-2) 3D gas-sensing patterns of the binary gas mixtures projected onto a representative 2D plot. The gas-sensing patterns are grouped by light blue colored regions, and the corresponding background relative humidity (R.H) levels are labeled. (a-3 (**I**)) Training accuracy and the training loss history from results using the pristine graphene; (**II**) confusion matrix of the pristine GFET. (a-4 (**I**)) Training accuracy and the training loss from results using the ALD-RuO_2_ GFET; (**II**) confusion matrix of the ALD-RuO_2_ GFET with 100% true values. (a-5) Normalized feature importance for the eight tested features. Reproduced with permission from [140], copyright 2020 Springer Nature.

Most recently, Hayasaka et al. [140] fabricated a highly selective sensor using pristine graphene and ALD-RuO_2_-based GFET devices with machine learning. In their proposed scheme, the measured V-shaped conductivity profiles were decoupled into four distinctive physical properties combined with other parameters. These four parameters were used as input feature vectors to classify different gases including electron mobility (*µ_e_*), hole mobility (*µ_h_*), ratio of the electron and hole concentration (*n_e/h_*), the ratio of the residual carrier, and charge impurity concentration (n*/n_imp_), represented in Equations (9)–(12), respectively [140].

(9) $$\mu_{e}=\frac{1}{C_{G}}\frac{\Delta \sigma_{e}}{\Delta V_{G}}$$

(10) $$n_{e/h}=\frac{C_{G}}{e}\left|V_{NP}\right|$$

(11) $$\mu_{h}=\frac{1}{C_{G}}\frac{\left|\Delta \sigma_{h}\right|}{\Delta V_{G}}$$

(12) $$\frac{n^{*}}{n_{imp}}=\frac{1}{20}\frac{h}{e^{2}}\sigma_{0}$$

where $C_{G}$ is the gate capacitance per unit area, $\Delta \sigma_{e}$ is the change in electron conductivity, $\Delta \sigma_{h}$ is the change in hole conductivity, $\Delta V_{G}$ is the change in gate voltage,$\;n_{h}$ is the hole concentration, e is the elementary charge, $V_{G}$ is the gate voltage, $V_{NP}$ is the gate voltage at the neutrality point (NP), h is Planck’s constant, and $\sigma_{0}$ is the minimum conductivity at the NP. The electron mobility and hole mobility turned out to be the most important features, with electron mobility having much of the information of the data. The confusion matrix displayed 100% correct values for ALD-RuO_2_-GFET, while pure graphene GFET confused some values with others [140] (see Figure 18).

A multilayer perceptron classifier with a feed-forward neural network architecture was implemented and trained using the 4D input feature vector of the two GFETs (pure graphene and ALD-RuO_2_). To avoid overfitting, the entire dataset was randomly shuffled in several ways and then separated via a stratified split, where 20% was reserved as the testing set and the remainder constituted the training set. The neural network model was trained for 40 epochs to obtain the maximum accuracy, and the time required for 40 epochs was 0.0519 s. The accuracies of the pristine GFET device and ALD-RuO_2_-GFET device were 96.2% and 100%, respectively. The cross-validation results indicated that the pristine GFET device had a mean accuracy of 95.4% and a standard deviation of 2.5%, whereas the ALD-RuO_2_-GFET device had a mean accuracy of 99.6% and a standard deviation of 0.8%. [140]. A comparison of FET devices with extracted features and output accuracies is summarized in Table 2.

### 3.3. Smart Breath Analyzers Using Machine Learning

**Figure 19:**
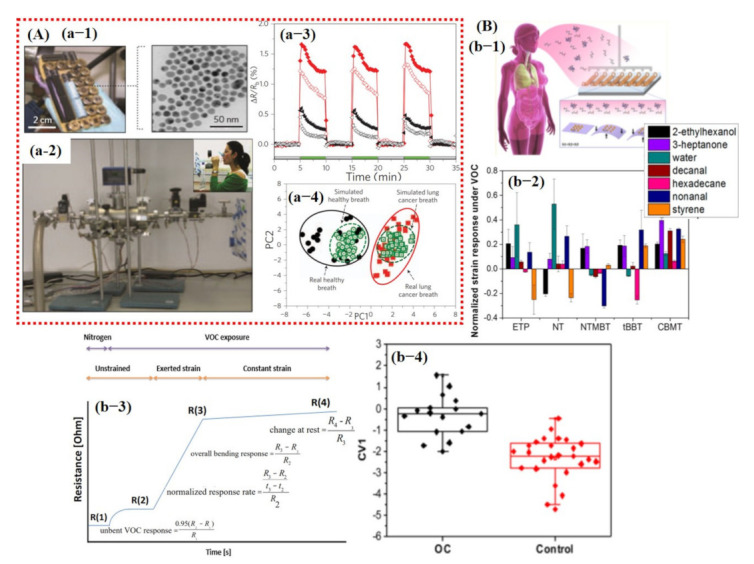
Smart breath analyzers using chemiresistive devices and machine learning. (**A**) Lung cancer detection using functionalized Au NPs from exhaled breath: (a-1) Optical image of sensor array; SEM image of Au NPs. (a-2) Breath sample collection and sensing setup. (a-3) Functionalized Au NPs sensor response towards healthy (filled symbol) and lung cancers patients (empty symbol). The significant change in response can be seen from the graph. (a-4) PCA of the dataset of real and simulated breath. Reproduced with permission from [146], copyright 2020 Nature. (**B**) Functionalized Au NPs onto flexible substrate was used to detect the ovarian carcinoma from exhaled breath. (b-1) Schematic diagram of fabricated sensor device with bending illustration upon exposure of any VOC. (b-2) Normalized strain response produced in different ligands under various VOCs. (b-3) Extracted feature graph for data processing. (b-4) Separation of the OC-positive and control groups (OC = ovarian cancer) using LDA. Reproduced with permission from [148], copyright 2015 ACS.

**Figure 20:**
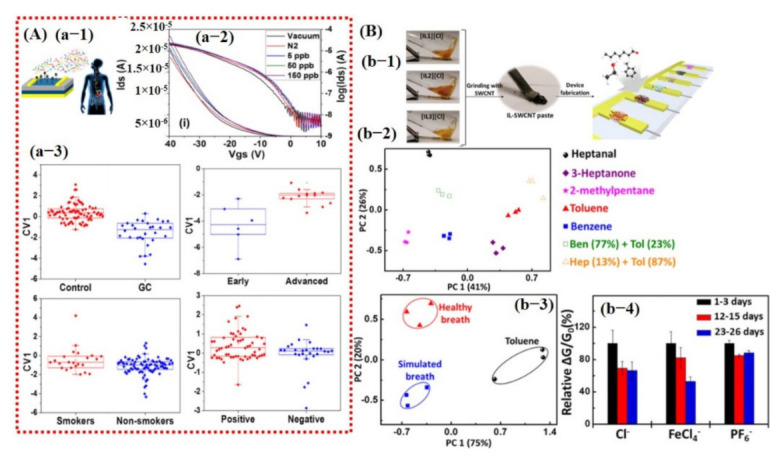
Smart breath analyzers using FET devices and machine learning: (**A**) Surface-modified single Si NW FET device for cancer detection: (a-1) Schematic diagram of fabricated sensor. (a-2) Variation in IDS curve upon exposure to the increasing concentrations of VOCs. (a-3) CV1 values resulting from DFA analysis of the breath samples gastric cancer vs. control, early stages vs. advanced stages, smokers vs. nonsmokers, H. pylori-positive vs. H. pylori-negative. Reproduced with permission from [141], copyright 2015 ACS. (**B**) Ionic liquid-functionalized CNT-based FET sensing array was used for detection of different VOCs: (b-1) Schematic diagram of a fabricated array. (b-2,3) PCA results towards different VOCs. (b-4) The stability of ionic liquid after several days. Reproduced with permission from [149], copyright 2018 ACS.

A multilayer perceptron classifier with a feed-forward neural network architecture was implemented and trained using the 4D input feature vector of the two GFETs (pure graphene and ALD-RuO_2_). To avoid overfitting, the entire dataset was randomly shuffled in several ways and then separated via awareness of personal health conditions demanding the development of safe, easy, and noninvasive disease diagnostic tools with great accuracy. Analysis of various VOCs concentration levels in exhaled breath samples opens up a new frontier in the medical sector due to its easy installation, cheap fabrication, and noninvasive diagnostic nature. However, the development of a small size breath analyzer with accurate and fast detection of a particular compound at a certain concentration level among 1000 VOCs is still a great challenge [143,144,145]. Enormous efforts have been made for the fabrication of a smart, small-size, economical, highly selective, and accurate breath analyzer using both chemiresistive and FET mechanisms. In fulfillment of such requirements, Haick et al.’s [141,146,147,148] research group made significant efforts and published a series of research articles using ligand-functionalized Au NPs. In 2009, lung cancer detection in exhaled breath was performed using functionalized Au NPs [146]. They built a sensing array using a surface modification of Au NPs (5 nm) with several organic ligands including dodecanethiol, decanethiol, 1-butanethiol, 2-ethylhexanethiol, hexanethiol, tert-dodecanethiol, 4-methoxy-toluenethiol, 2-mercaptobenzoxazole, and 11-mercapto-1-undecanol. Interestingly, sensors functionalized with 4-methoxy-toluenethiol, and 2-mercaptobenzoxazole/11-mercapto-1-undecanol showed detection limits of 2–10 ppb on exposure to acetaldehyde (a promising VOC for lung cancer) and formaldehyde (a promising VOC for breast cancer). Furthermore, 2-mercaptobenzoxazole–Au NPs (red diamonds) and tert-dodecanethiol–Au NPs (black triangles) displayed a significant difference in responses towards healthy and lung cancer patients, suggesting them as a promising candidate for the detection of lung cancer [146] (see Figure 19A). The response data obtained from healthy and lung cancer breath samples were then processed through PCA for classification. The PCA graph shown in Figure 19A demonstrates highly discriminated results without any overlapping. Features for PCA analysis and training of any classifier were not discussed in their article [146]. Likewise, in 2010, the same group developed ligand-modified Au NP sensing array for the detection of lung, breast, colorectal [147], and prostate cancers from exhaled breath. Their PCA graph illustrates a discriminative signature pattern for each kind of cancer disease with no overlapping on healthy samples. They mentioned that this is an attempt for the development of a cost-effective, easy-to-use, portable, and non-invasive diagnostic tool for detecting lung, breast, colorectal, and prostate cancers through a single breath test [147]. In 2015, a surface-modified Si NW-based FET sensor was developed for the diagnosis of gastric cancer. Instead of sensing arrays, they developed a single sensor-based FET device to selectively detect gastric cancer-related VOCs [141]. For data processing, three features were extracted from the I_DS_ vs. V_GS_ curves as a function of the exposure time towards the targeted VOCs: the threshold voltage (V_th_); hole mobility (μ_h_), inferred from the linear part of the curve; and the current at zero applied gate voltage (I_DS_ @ V_GS_ = 0), as a representative subthreshold current (see Figure 20A) [141]. The dataset of each analysis was divided into training and validation sets. A total of 75% of each group was selected randomly for the training set, and 25% of each group were left out for testing. Leave-one-out CV was conducted to train the DFA for the classification of the number of true-positive, true-negative, false-positive, and false-negative predictions. The training set using only one sensor (S1) showed 87% sensitivity, 81% specificity, and 83% accuracy [141]. Kahen et al. [148] developed ligand-functionalized Au NP sensing array on a flexible substrate for diagnosis of ovarian carcinoma from exhaled breath. In order to generate distinctive responses from each target compound and to increase the number of features for data processing along with resistance change, they also observed the bending state of the substrate upon absorption of the particular compound. The extracted features from the response curve are shown in Figure 19B. Discriminant factor analysis (DFA) was used with the leave-one-out CV method to find the sensitivity, specificity, and accuracy of each sensor using bending-related features from only one sensor. Their DFA result showed 83.4% sensitivity, 80.8% specificity, and 81.8% accuracy, which was comparable with previously published results [148] (see Figure 19B). 

In 2018, Sujono et al. [150] reported an e-nose for asthma diagnosis in exhaled breath that can predict the related VOC detection accuracy using SVM. The e-nose consists of seven commercial sensors including CO, H_2_, NOx, H_2_S, NH_3_, VOCs, and CO_2_. A window of dynamic response curve (30 to 49 s) was used as a feature to train the SVM classifier. Their system can successfully distinguish between healthy and asthma subjects with an accuracy rate of 89.5% [150].

In the same year, Park et al. [149] demonstrated an ionic liquid-functionalized carbon nanotubes (CNTs) sensing array for detection of exhaled breath-related VOCs. They developed a new platform for the selective and sensitive detection of VOCs by exploring the influence of cation and anion and identifying swelling as one of the sensing mechanisms. Nine sensors were built by surface modification of CNTs for the detection of 3-heptanone, heptanal, 2-methylpentane, benzene, and toluene. Figure 20B shows the PCA scores and it indicates the excellent discriminating ability of the sensors among different VOCs. However, the extracted features for obtaining PCA graphs were not discussed in their work. Ionic liquid demonstrates promising capabilities in detecting several VOCs, with distinguished transient patterns eventually leading to the development of highly selective VOC sensors using machine learning [149].

Although machine learning is treated as one of the major contributors in building smarter and selective sensors, there are still great obstacles in terms of developing economical, miniaturized, low-powered smarter sensors with the ability to accurately discriminate among different gas classes and concentration levels under various environmental circumstances (dry and humid conditions). Most of the metal oxide gas sensors show sensitivity to different gases at different concentrations, with minute change in their response curve. Therefore, it is essential to accurately detect and categorize the class of gas with its concentration level through proper machine learning algorithms in order to avoid any unwanted false positives. Further improvements in different aspects are required for successful implementation in real-world application; for instance, the development of a sensor with a low aging effect that works properly under different environmental conditions with good repeatability, and the implementation of different data processing algorithms to accurately train a model with minimum possible dataset examples and low power consumption. In the early days, larger datasets under all circumstances were required with maximum possible examples to enhance the training probability rate of the final statistical model. Several e-nose systems with multiple arrays of sensors were utilized to obtain the large datasets with all sensing examples. Recently, this problem has been partially resolved by introducing the k-fold cross-validation technique in which the model is trained and tested on all available examples; thus, smaller datasets can be used to accurately train a model. However, in this case, the signal processing unit may consume higher power due to the large number of iterations for the model training. The power consumption may be compensated through the sensory unit by reducing the arrays of sensors to a single sensor. Machine learning is successfully able to selectively detect various chemical compounds using a single sensing device (low-power consumption) with the k-fold cross-validation (smaller datasets) technique. However, the complex, expensive, and large hardware setups of smart sensors are pressing the need for developing nano/micro-sized sensory and signal processing units to accomplish the whole machine learning process on a single MEMS/NEMS chip for the miniature and portable smart system. 

## 4. Conclusions and Outlooks

This review covers the recent state-of-the-art advancement in the field of gas sensing using different techniques and their limitations and solutions using machine learning tools. The article is divided into two parts: The first part emphasizes the recent progress in the field of gas sensing, their different performance improvement methods, and current challenges. The second part highlights the significance of machine learning as a potential approach to tackle these limitations. Moreover, recent development in smart sensors and breath analyzers using machine learning has been also discussed in details. 

For the implementation of a sensor in a real-world application, a sensor must possess several essential sensing performance features including excellent sensitivity, fast response/recovery time, repeatability, long-term stability (in humid and high-temperature environments), and selectivity. Different performance-enhancing methods have been discussed, such as composition/hybridization, doping of heteroatom, p–n junction formation, and core–shell structures. According to the base material, we categorized gas sensors into three types: (1) graphene-based, (2) TMDC-based, and (3) semiconductor/metal oxide-based. Their computational studies were also covered to realize the major performance-enhancing factors. Furthermore, performance improvement methods for all three categories with their advantages and drawbacks were detailed. It was concluded that performance-enhancing methods certainly improve sensitivity, response/recovery time, repeatability, the limit of detection, and operating temperature. However, excellent selectivity and long-term stability in different environments are still great challenges. Therefore, it is suggested that with the development of novel material, efficient data processing techniques using machine learning are greatly needed in order to tackle selectivity and long-term stability.

Machine learning has been extensively used in building highly selective smart gas sensors and breath analyzers. Data processing is a major contributor since the success of the machine learning process relayed on it. It aims to extract robust feature information from the dynamic sensor response, which can represent the unique “fingerprint” patterns for a particular gas to ensure the effectiveness of the subsequent pattern recognition algorithm. This article discussed the state of the art of chemiresistive and FET-based smart gas sensors along with the most important features, which can be derived from the original dynamic response. Moreover, recent evolution in breath analyzers using machine learning has been also covered in this article.

Machine learning shows great potential in solving critical issues related to chemical gas sensors and plays a significant role in building smart sensors with improved sensing abilities, especially selectivity and long-term stability. However, they are complex, expensive, consume high power, and require a large hardware setup for their implementation in a realistic application. This presses the need for developing nano/micro-sensors along with signal processing and machine learning algorithm units on a single flexible substrate using MEMS/NEMS technology for the realization of miniature and portable electronics/electrical revolutionary smart world. 

## Figures and Tables

**Table 1:** A comparison of some of the reported extracted features and output accuracies for chemiresistive devices.

Sensor	Material	Objective	Features	Data	Data Processing	Model	Accuracy
Resistive [131]	WO_3_ thin film	O_3_ concentration classification	Static: ^a^ R_gas_/R_air_	NM	PCA * TV = 77%	NM	NM
			Dynamic: area under response time curve		PCA * TV = 99.62%		
Resistive [123]	Unmodified graphene	Identification of chemical compounds	^b^ ∆R	20 cycles/chemical	PCA * TV for ^b3^ CDC = 98.65%	K-NN	95%
			^b1^ A_Resp_			SVM	95%
			^b2^ A_Recov_			RF	96%
			A_Resp_/A_Recov_			LDA	92%
Resistive [132]	Metal oxide sensors	Classification of ethanol and acetone	NM	300 measurements	PCA * TV = 75.5%	LDA	76.4%
Resistive [135]	Functionalized CNTs	Food classification	CWT FFT	12 cycles/food sample	PCA	^c^ f-RF	91%
			Time response data			K-NN	73%
Resistive [124]	NiO NWs	Classification of hazardous gases	Response value at different ^d^ OT (200–400 °C)	8 measurements	PCA: * TV = 99.8%	LR	100%
						RF	100%
						SVM	100%
Resistive [125]	Commercial VOC sensors	Discrimination among VOCs	Normalized response value	NM	PCA:* TV = 82%	LDA	78%
Resistive [129]	Graphene nanoribbons	Chemicals detection and classification	^e^ $\;N=S_{i}/S_{avg}$	3–5 measurements/chemical	NM	LDA	100%
Resistive [126]	SnO_2_ hollow spheres	VOC detection	^d^ OT	64 measurements	NM	RF	85.93%
			Sensitivity			MLP	76.56%
			Response time			SVM	82.81%
			Recovery time			NB	68.75%

a = ratio between resistance values before and after gas exposure, b = change in resistance before and after gas exposure, b1 = area under response time curve, b2 = area under recovery time curve, b3 = chemically diverse set, b4 = substituted benzene set, c = featured trained random forest, d = operating temperature, e = normalized response where $S_{i}$ is the response of an individual sensors and $S_{avg}$ is the average response across the array of n sensors, * = total variance, NM = not mentioned.

**Table 2:** A comparison of some of the reported extracted features and output accuracies for FET devices.

Sensor	Material	Objective	Features	Data	Model	Accuracy
FET [141]	Functionalized Si NWs	Cancer diagnosis	V_th_	Data from 77 volunteers	DFA	85%
			μ_h_			
			I_DS_@V_GS_ = 0			
^a^ CNFET [139]	Metal/CNTs	Purine classification	Slop variance	12 measurements/chemical	SVM	93.4%
			V_th_			
			Changes in conductance at various V_G_ values			
^b^ ISFET [142]	HfO_2_ layer	pH and light intensity	Normalized response at various biasing voltages	1920 measurements from 32 sensors	SVM	100%
					^b1^ BPNN	100%
FET [140]	^c^ GFET	Water, methanol, and ethanol detection	$\mu_{e}$	100 measurements/chemical	Multi-layered ANN	96.2%
			$n_{e/h}$			
	^c1^ ALD-RuO_2_-GFET		$\mu_{h}$			100%
			$\frac{n^{*}}{n_{imp}}$			

a = carbon nanotube field effect transistor, b = ion-sensitive field effect transistor, b1 = back propagation neural network, c = graphene field effect transistor, c1 = atomic layer deposition-grown RuO_2_ thin film field effect transistor.

## Appendix A

Here, we present some background information on the PCA technique. PCA aims to reduce redundancy in a large dataset through diagonalization of the correlation matrix of the original dataset for the identification of the most important parameters in defining the actual degree of freedom or response of a system. The variance and covariance among the data values can be obtained by taking the dot product of the dataset with its transpose, as shown in Equation (A1)

(A1) $$C_{X}=\frac{1}{n-1}XX^{T}$$

where $C_{X}$ is the correlation matrix of dataset X and n is the number of variables.

(A2) $$X=\left(\begin{array}{c} {\underset{x}{\to }}_{a}\\ {\underset{y}{\to }}_{a}\\ {\underset{x}{\to }}_{b}\\ {\underset{y}{\to }}_{b}\\ {\underset{x}{\to }}_{c}\\ {\underset{y}{\to }}_{c} \end{array}\right)$$

(A3) $$C_{X}=\left(\begin{array}{cccccc} V_{x_{a}x_{a}} & CV_{x_{a}y_{a}} & CV_{x_{a}x_{b}} & CV_{x_{a}y_{b}} & CV_{x_{a}x_{c}} & CV_{x_{a}y_{c}}\\ CV_{yax_{a}} & V_{y_{a}y_{a}} & CV_{y_{a}x_{b}} & CV_{y_{a}y_{b}} & CV_{yax_{c}} & CV_{y_{a}y_{c}}\\ CV_{x_{b}x_{a}} & CV_{x_{b}y_{a}} & V_{x_{b}x_{b}} & CV_{x_{b}y_{b}} & CV_{x_{b}x_{c}} & CV_{x_{b}y_{c}}\\ CV_{y_{b}x_{a}} & CV_{y_{b}y_{a}} & CV_{y_{b}x_{b}} & V_{y_{b}y_{b}} & CV_{y_{b}x_{c}} & CV_{y_{b}x_{c}}\\ CV_{x_{c}x_{a}} & CV_{x_{c}y_{a}} & CV_{x_{c}x_{b}} & CV_{x_{c}y_{b}} & V_{x_{c}x_{c}} & CV_{x_{c}y_{c}}\\ CV_{y_{c}x_{a}} & CV_{y_{c}y_{a}} & CV_{y_{c}x_{b}} & CV_{y_{c}y_{b}} & CV_{yc_{a}x_{c}} & V_{y_{c}y_{c}} \end{array}\right)$$

where V is the variance among the same variable and CV is the covariance among the different variables. Put simply, the diagonals are the variance measure (V) and off-diagonals are the covariance values (CV). The small off-diagonal values indicate the statistical independence among the variables. However, if these values come out as big numbers then this indicates redundancy in the system data, which can be eliminated through the diagonalization of the whole matrix. This means that all off-diagonal values will be reduced to zero and the critical information of the system will be restricted to the first and uppermost diagonal value (eigenvalue). In some cases, it may go up to the second and third eigenvalue, which are known as first, second, and third PCA scores of a system. Finally, to obtain the PCA results, one can always decompose the $C_{X}$ in the eigenvalues and vectors due to its symmetric nature, as shown in Equation (A4):

(A4) $$C_{X}=XX^{T}=S\Omega S^{T}$$

where S is the matrix of eigenvectors and Ω is the eigenvalue diagonal matrix of the $VC_{X}$. The eigenvalue diagonal matrix (Ω) can be obtained by taking the dot product of the original dataset with the transpose of the eigenvector matrix of the $VC_{X}$, as shown in Equation (A5):

(A5) $$Y=XS^{T}$$

(A6) $$C_{Y}=\frac{1}{n-1}YY^{T}$$

where Y is matrix and $C_{Y}$ is a correlation matrix. After calculation and replacing the $Y=XS^{T}$ in Equation (A6), the final diagonal eigenvalue matrix can be obtained to determine the PCA scores of the system:

(A7) $$C_{Y}=S^{T}S\Omega S^{T}S=\Omega$$

### Appendix A.1. Chemiresistive Gas Sensors

A chemiresistive gas sensor is a device that detects different levels of gas concentrations and shows the change in resistance at the output. The increase or decrease in the resistance depends on the nature of the sensing material and target gases (oxidizing/reducing). The oxidizing gases trap while the reducing gases donate the electrons during the reaction process at the sensing material surface.

### Appendix A.2. MOSFET Gas Sensors

A MOSFET device consists of three terminals: drain (D), source (S), and gate (G). In MOSFETs, the drain to source current (IDS) flows through the thin layer of metal oxide-sensing material. The device shows the change in IDS values after the adsorption of target gas molecules on the sensing material surface.

### Appendix A.3. Smart Gas Sensors

A smart gas sensor is a device that efficiently and accurately detects particular gas traces among several other gases through a unique pattern recognition method using machine learning.

### Appendix A.4. Breath Analyzers

A breath analyzer is a non-invasive device that is used to precisely detect a particular gas or VOC at a specific concentration level among thousands of other VOCs in human breath for accurate disease diagnosis.

### Appendix A.5. Selectivity

Selectivity can be defined as the ability of a sensor to accurately detect the traces of a particular gas among several other gases in the environment.

### Appendix A.6. Classifiers

Classifiers are used to accurately discriminate among different classes. Firstly, unsupervised data processing techniques such as PCA and LDA are used to generate unique signatures for each class, and then a classifier is trained on this processed data to accurately predict different classes from an unseen dataset.

## Nomenclature

MOSFET	Metal-oxide-semiconductor field effect transistors
SPR	Surface plasmon resonance
rGO	Reduced graphene oxide
CNTs	Carbon nanotubes
2D	Two-dimensional
3D	Three-dimensional
TMDCs	Transition metal dichalcogenides
MOx	Metal oxides
VOCs	Volatile organic compounds
e-noses	Electronic noses
FFT	Fast Fourier transform
CWT	Continuous wavelet transform
DWT	Discrete wavelet transform
PCA	Principal component analysis
LDA	Linear discriminant analysis
KNN	*k*-nearest neighbors
CART	Classification and regression trees
NB	Gaussian naïve Bayes
SVM	Support vector machines
RF	Random forest
DFT	Density function theory
DOS	Density of state
VSCs	Volatile sulfur compounds
LOD	Limit of detection
LPWN	Low-power wireless network
M2M	Machine to machine
LR	Logistic regression
R_air_	Sensor resistance in air
R_gas_	Sensor resistance after gas exposure
ΔR	Difference between R_air_ and R_gas_
A_Resp_	Area under response time curve
A_Recov_	Area under recovery time curve
Cavitand	A container shaped molecule with a cavity
Tsfresh	A tool for feature extraction
FETs	Field effect transistors
CV	Cross-validation
ANN	Artificial neural networks
DFA	Discriminant factor analysis
MEMS	Micro-electromechanical systems
NEMS	Nano-electromechanical systems
